# Supplementary material for: The future of feedback: Motivating performance improvement through future-focused feedback
Source: PLoS One. 2020 Jun 19;15(6):e0234444. doi: 10.1371/journal.pone.0234444 (PMC7304587; doi:10.1371/journal.pone.0234444)
Supplement: S1 Text — (PDF) [file pone.0234444.s001.pdf]

The future of feedback: Motivating performance improvement

Jackie Gnepp, Joshua Klayman, Ian O. Williamson, Sema Barlas

S1 Text. Study 1 instructions and survey

Managerial Psychology Poll

Hello!
This first class assignment is a short, on-line survey. We estimate that it will require about 10 minutes to complete.

The survey will ask for your name, but only so we can give you credit for having completed this assignment. Your name will be separated from your responses to the survey, and all responses will remain anonymous. In class we will discuss aggregate results only; no individual answers will be reported.

In order to keep the survey short, we are dividing different questions up among different people. To allow us to divide people up evenly, please answer the question below about the **last two digits** of your **home** phone number. Even though this seems like a meaningless question, we need you to provide the real answer in order to divide people up evenly.

Click on the one answer below that applies to you. That will automatically take you to the survey. Thanks!

The last two digits of my home phone number are

00 to 24 **25 to 49** **50 to 74** **75 to 99**

If you have any questions or problems, let us know: joshk@uchicago.edu

Managerial Psychology

This survey asks you to describe and evaluate a recent work-related incident that involved feedback. We ask that you indicate your name, but only so we can tell who has completed the survey. We will not be able to associate your name with your answers and we will not reveal any individual answers, only aggregate patterns.

Your family name:

Your given name:

Which class are you in?

- Section C, 27 June - 2 July
- Section B, 4 - 9 July

Which program are you in?

- AXP
- EXP
- XP

A. Please think of a recent incident about which you gave negative feedback. Briefly describe the incident and the feedback.

B. Rate the accuracy of the feedback, from 0% to 100%

0 5 10 15 20 25 30 35 40 45 50 55 60 65 70 75 80 85 90 95 100

C. How qualified were you to give the feedback? Rate from 0 = unqualified to 10 = completely qualified:

0 1 2 3 4 5 6 7 8 9 10

D. Looking back now at the incident, please assign a percentage to each of the following four causes, such that the four of them together **sum to 100%**.

% due to abilities he or she lacked

0 5 10 15 20 25 30 35 40 45 50 55 60 65 70 75 80 85 90 95 100

% due to the amount of effort he or she put in

0 5 10 15 20 25 30 35 40 45 50 55 60 65 70 75 80 85 90 95 100

% due to the nature of what he or she had to do

0 5 10 15 20 25 30 35 40 45 50 55 60 65 70 75 80 85 90 95 100

% due to bad luck

0 5 10 15 20 25 30 35 40 45 50 55 60 65 70 75 80 85 90 95 100

Click the "Submit" button to record your answers.

Submit
