# Supplementary material for: The future of feedback: Motivating performance improvement through future-focused feedback
Source: PLoS One. 2020 Jun 19;15(6):e0234444. doi: 10.1371/journal.pone.0234444 (PMC7304587; doi:10.1371/journal.pone.0234444)
Supplement: S2 Text — (DOCX) [file pone.0234444.s002.docx]

**The future of feedback: Motivating performance improvement**

Jackie Gnepp, Joshua Klayman, Ian O. Williamson, Sema Barlas

**S3 Text. Study 2 instructions.**

**Background:** The DeltaCom Corporation is a multi-million dollar developer, manufacturer and marketer of telecommunications and switching equipment. DeltaCom originally started out as a small group of engineers, led by Dominique Hernandez, who were disillusioned by the bureaucracies associated with the telecommunications giants (e.g., Telstra). Working out of the basement of an old warehouse in Sydney, the team of young, entrepreneurial engineers grew the business from start-up in early 1990’s to a respected and recognized telecommunications leader in New South Wales, Queensland and Victoria. Much of the company’s success is directly related to the establishment and maintenance of strong partnerships with large multi-national enterprises. The expanding world economy and optimism of the early years of the new millennium also fueled a surge in new customers. Given its tremendous financial success, it is not surprising that DeltaCom continues to grow. To hold onto its gains, DeltaCom instituted its Strategy for the New Economy in 2004, focused on customer satisfaction and customer retention. Naturally, DeltaCom has experienced a certain amount of turmoil and turnover of personnel as might be expected for a company experiencing rapid growth. Nonetheless, consistent with the corporate vision of becoming a “world-class telecommunications leader,” DeltaCom has recently been positioning itself to expand to and “dominate” Western Australia.

[For District Manager role]

**Your role:** For the purposes of this exercise, you are Taylor Devani, a District Manager (Melbourne Territory). Currently, you are one of 5 district managers who report to Chris Sinopoli, a newly hired Regional Manager (Southeast Region). Chris was hired to replace your previous boss, Linn Woo, an organizational superstar who left the company to pursue other interests. You had hoped to be promoted into that position when your former boss left, and were disappointed to learn that the person hired wasn’t even from inside the company! Now, however, you are being considered for a Regional Manager position in Western Australia, a very exciting opportunity.

In a short while, you will meet with Chris Sinopoli to discuss your candidacy for this promotion. If you are promoted, you will inherit a broader scope of authority six months from now. **Chris called this meeting specifically to give you performance feedback regarding the managerial skills needed to succeed in this new position.** Unfortunately, Chris really has not had the opportunity to get fully acquainted with you. Thus, to help you prepare for this feedback session, you have gathered some basic information from your personnel file, along with some updated information concerning your recent activities.

**Your task: You have 15 minutes to review the enclosed information, and an additional 5 minutes to complete the short questionnaire following. You will then have 20 minutes to meet with Chris and discuss performance issues. After the meeting, you will complete a questionnaire labeled Part 2. Please stay in role during the entire exercise.**

[For Regional Manager role]

**Your role:** For the purposes of this exercise, you are Chris Sinopoli, a newly hired Regional Manager (Southeast Region). You were hired to replace Linn Woo, an organizational superstar who left the company to pursue other interests. Five District Managers now report to you, including Taylor Devani (Melbourne Territory). Taylor had hoped to be promoted into the position you now hold, and was disappointed to learn that the person hired wasn’t even from inside the company! Now, however, Taylor is being considered for a Regional Manager position in Western Australia, a very exciting opportunity.

In a short while, you will meet with Taylor Devani to discuss Taylor’s candidacy for this promotion. If promoted, Taylor will inherit a broader scope of authority six months from now. **You called this meeting specifically to give Taylor performance feedback regarding the managerial skills needed to succeed in this new position.** Unfortunately, you really have not had the opportunity to get fully acquainted with Taylor. Thus, to help you prepare for this feedback session, your secretary has gathered some basic information from Taylor’s personnel file, along with some updated information concerning Taylor’s recent activities.

**Your task: You have 15 minutes to review the enclosed information, and an additional 5 minutes to complete the short questionnaire following. You will then have 20 minutes to meet with Taylor and discuss performance issues.**  **Focus on Taylor’s weaknesses as a manager -- those aspects of performance Taylor must change to achieve future success if promoted. After the meeting, you will complete a questionnaire labeled Part 2. Please stay in role during the entire exercise.**

| **Personnel File for Taylor Devani**  **Date of Hire: June 2002** |
| --- |
| **Position: District Manager, Melbourne** |

**Administrative**

- Received an MBA from the Australian Graduate School of Management, 2001
- Previously employed as a sales representative by Central Telecom, a leading competitor
- Hired on with DeltaCom as a District Manager for the Melbourne territory
- Within about 1 year as District Manager, had 5 of 9 employees turnover due to promotion (1 employee) and voluntary resignation (4 employees)
- Within about 1 year, vaulted the district from average to one of the best in overall sales performance. More than doubled sales.
- Exit interviews conducted with the employees who left the district yielded several complaints, as well as comments contending that Taylor was “moody, tyrannical, and obsessive.”
- Recommended the termination of Jan Haskins in January, 2005. Termination completed.
- 360-degree feedback results from February, 2009 (surveys were distributed in October 2008) place Taylor well above company average in terms of coaching and training behavior, however there was low agreement among respondents. Open ended comments indicated that Taylor was “driven”; “a real mover, willing to push us over the top”; a gigantic step toward my personal career mobility!”; “masterful motivator”; “If you look up demanding in the dictionary, you’ll see his picture”; and “out to rule the world.”
- Recommended the termination of Hank Thibodeaux in February, 2009. Termination pending documentation. (Note: Taylor hired Hank to replace one of the representatives who left after Taylor’s arrival. At that time, Hank’s sales performance was similar to that of Jan Haskins – highly consistent and well below average, about $40 K per month. Taylor argued at that time that Hank was going to do well as a representative, and that his lagging sales were due to his lack of experience and his assignment to a highly entrenched area.)
- Instituted probationary status for Terry Webb in January 2009. Pending re-evaluation in 6 months. (Note: Terry was one of the original nine employees Taylor inherited when Taylor took over the district, and is now one of the three remaining survivors. His sales have improved at a moderate rate since Taylor’s arrival and have been well above the company’s average for quite some time, at least 3 quarters. Terry’s customer satisfaction ratings have been consistently high.).

**Rewards/Recognition**

- Recipient of the “Pinnacle Award” in December, 2007 (DeltaCom’s top sales/citizenship award)
- During the annual employee recognition banquet held in September 2008, Taylor received three of the five awards available to the company’s District Managers. The awards received cited the excellence of Taylor’s work as a District Manager in (1) leading the district to achieve the highest annual sales in Australia; (2) driving the sales force to attain the greatest annual increase in new customers in Australia; and (3) maximizing the contributions of a sales force to attain globally competitive levels of performance. In addition, Taylor and one of the sales representatives received a certificate and special commendation for making the largest sell to a single customer in the company’s history – a deal which yielded a contract worth over $1.2 M in product going to a single customer over the period of one year.

**Key Performance Indicators**

|  | June 2008 | June 2007 | June 2006 | June 2005 | June 2004 | June 2003 | **Co. Avg** |
| --- | --- | --- | --- | --- | --- | --- | --- |
| % Orders Retained | 74 | 76 | 78 | 81 | 78 | 80 | **86** |
| % Increase in Customers | 25 | 23 | 29 | 17 | 23 | 21 | **14** |
| % Change in Sales | 16 | 15 | 11 | 13 | 12 | 20 | **8** |
| Total Sales in Millions | 3.5 | 3.1 | 2.7 | 2.4 | 2.1 | 1.9 | **2.3** |
| % Customer Satisfaction | 75 | 80 | 78 | 80 | 87 | 83 | **91** |

**Notes of 2008 appraisal feedback interview by Linn Woo**

I met with Taylor to appraise strengths and weaknesses in the current job and look ahead to future assignments. I congratulated Taylor on winning practically every sales award the firm offers, a testament to Taylor’s drive, ambition, and ability to motivate many salespeople. Taylor was especially pleased to hear of my promotion recommendation and that President Dominique Hernandez also spoke highly about Taylor. Taylor is aware that many in the rank and file believe that Taylor is one of the best candidates for a future Regional Manager position. I reviewed several areas of developmental need that I think Taylor should address, including:

- Lack of appreciation for employees as human capital investments (i.e., Taylor only sees them as vehicles for bringing in sales dollars);
- A driven, demanding, and intolerant managerial style;
- An inability to coach employees who are not like Taylor (to put it bluntly, Taylor only succeeds in facilitating the performance of those employees who are driven, highly aggressive, self-absorbed, headstrong, and very focused on their own career);
- A limited customer service orientation – Taylor’s focus is largely on generating new customers. Although Taylor is successful at attracting new clientele, Taylor is much less effective at retaining them.

Taylor did not fully accept this developmental feedback, arguing that the most important characteristic of an outstanding sales firm is having hard working, resilient people, capable of aggressively pursuing sales. Maybe some of them are a little obnoxious, but they almost have to be in order to get the new sales. There is no room for being soft or sentimental in this line of work, and a focus on the bottom line, Taylor argues, is the key to sales success. Taylor makes no apology for the employees who left or were let go, saying those were the ones who weren’t willing to go the extra distance to obtain the new sales.

**(Signed) Linn Woo**

Please continue to the questions on the next page…District Manager, Part 1

Please rate Taylor Devani’s **level of job performance** on each of the following factors (1=very low performer to 7=very high performer):

Very low

performer

Low

performer

High

performer

Very high

performer

| Sales Performance | 1 | 2 | 3 | 4 | 5 | 6 | 7 |
| --- | --- | --- | --- | --- | --- | --- | --- |
| Customer Retention | 1 | 2 | 3 | 4 | 5 | 6 | 7 |
| Customer Satisfaction | 1 | 2 | 3 | 4 | 5 | 6 | 7 |
| Ability to manage and coach employees | 1 | 2 | 3 | 4 | 5 | 6 | 7 |

Please rate how **important** each of these factors is for Taylor Devani’s job performance at DeltaCom (1=not important to 7=very important):

Not

important

Somewhat

important

Important

Very

important

| Sales Performance | 1 | 2 | 3 | 4 | 5 | 6 | 7 |
| --- | --- | --- | --- | --- | --- | --- | --- |
| Customer Retention | 1 | 2 | 3 | 4 | 5 | 6 | 7 |
| Customer Satisfaction | 1 | 2 | 3 | 4 | 5 | 6 | 7 |
| Ability to manage and coach employees | 1 | 2 | 3 | 4 | 5 | 6 | 7 |

Please continue to the next page…

Please give your opinion about the **causes of Taylor Devani’s successes** by assigning a percentage to each of the following four causes, such that the four causes together **sum to 100%**.

% due to Taylor’s abilities and personality

| □  0 | □  5 | □  10 | □  15 | □  20 | □  25 | □  30 | □  35 | □  40 | □  45 | □  50 | □  55 | □  60 | □  65 | □  70 | □  75 | □  80 | □  85 | □  90 | □  95 | □  100 |
| --- | --- | --- | --- | --- | --- | --- | --- | --- | --- | --- | --- | --- | --- | --- | --- | --- | --- | --- | --- | --- |

% due to the amount of effort and attention Taylor applied

| □  0 | □  5 | □  10 | □  15 | □  20 | □  25 | □  30 | □  35 | □  40 | □  45 | □  50 | □  55 | □  60 | □  65 | □  70 | □  75 | □  80 | □  85 | □  90 | □  95 | □  100 |
| --- | --- | --- | --- | --- | --- | --- | --- | --- | --- | --- | --- | --- | --- | --- | --- | --- | --- | --- | --- | --- |

% due to Taylor’s job responsibilities, DeltaCom’s expectations, and the resources provided

| □  0 | □  5 | □  10 | □  15 | □  20 | □  25 | □  30 | □  35 | □  40 | □  45 | □  50 | □  55 | □  60 | □  65 | □  70 | □  75 | □  80 | □  85 | □  90 | □  95 | □  100 |
| --- | --- | --- | --- | --- | --- | --- | --- | --- | --- | --- | --- | --- | --- | --- | --- | --- | --- | --- | --- | --- |

% due to chance and random luck

| □  0 | □  5 | □  10 | □  15 | □  20 | □  25 | □  30 | □  35 | □  40 | □  45 | □  50 | □  55 | □  60 | □  65 | □  70 | □  75 | □  80 | □  85 | □  90 | □  95 | □  100 |
| --- | --- | --- | --- | --- | --- | --- | --- | --- | --- | --- | --- | --- | --- | --- | --- | --- | --- | --- | --- | --- |

**PLEASE CHECK: Do the above four numbers add to 100%? If not, please revise.**

Please give your opinion about the **causes of Taylor Devani’s failures** by assigning a percentage to each of the following four causes, such that the four causes together **sum to 100%**.

% due to Taylor’s abilities and personality

| □  0 | □  5 | □  10 | □  15 | □  20 | □  25 | □  30 | □  35 | □  40 | □  45 | □  50 | □  55 | □  60 | □  65 | □  70 | □  75 | □  80 | □  85 | □  90 | □  95 | □  100 |
| --- | --- | --- | --- | --- | --- | --- | --- | --- | --- | --- | --- | --- | --- | --- | --- | --- | --- | --- | --- | --- |

% due to the amount of effort and attention Taylor applied

| □  0 | □  5 | □  10 | □  15 | □  20 | □  25 | □  30 | □  35 | □  40 | □  45 | □  50 | □  55 | □  60 | □  65 | □  70 | □  75 | □  80 | □  85 | □  90 | □  95 | □  100 |
| --- | --- | --- | --- | --- | --- | --- | --- | --- | --- | --- | --- | --- | --- | --- | --- | --- | --- | --- | --- | --- |

% due to Taylor’s job responsibilities, DeltaCom’s expectations, and the resources provided

| □  0 | □  5 | □  10 | □  15 | □  20 | □  25 | □  30 | □  35 | □  40 | □  45 | □  50 | □  55 | □  60 | □  65 | □  70 | □  75 | □  80 | □  85 | □  90 | □  95 | □  100 |
| --- | --- | --- | --- | --- | --- | --- | --- | --- | --- | --- | --- | --- | --- | --- | --- | --- | --- | --- | --- | --- |

% due to chance and random luck

| □  0 | □  5 | □  10 | □  15 | □  20 | □  25 | □  30 | □  35 | □  40 | □  45 | □  50 | □  55 | □  60 | □  65 | □  70 | □  75 | □  80 | □  85 | □  90 | □  95 | □  100 |
| --- | --- | --- | --- | --- | --- | --- | --- | --- | --- | --- | --- | --- | --- | --- | --- | --- | --- | --- | --- | --- |

**PLEASE CHECK: Do the above four numbers add to 100%? If not, please revise.**

Please continue to the next page…

Please print your name: _________________

The name of the person playing Regional Manager, Chris Sinopoli: _________________

Please tear off the green pages and place them in the envelope provided. When you are ready, you may begin your 20-minute meeting with Chris to discuss performance issues. After the meeting, you will complete a questionnaire labeled Part 2. Please do NOT complete Part 2 until ***after*** the 20-minute meeting. Please stay in role during the entire exercise.
