# Supplementary material for: The future of feedback: Motivating performance improvement through future-focused feedback
Source: PLoS One. 2020 Jun 19;15(6):e0234444. doi: 10.1371/journal.pone.0234444 (PMC7304587; doi:10.1371/journal.pone.0234444)
Supplement: S3 Text — (DOCX) [file pone.0234444.s003.docx]

**The future of feedback: Motivating performance improvement**

Jackie Gnepp, Joshua Klayman, Ian O. Williamson, Sema Barlas

**S4 Text. Study 2 pre-discussion questionnaire – Regional Manager.**

Regional Manager, Part 1

Please rate Taylor Devani’s **level of job performance** on each of the following factors (1=very low performer to 7=very high performer):

Very low

performer

Low

performer

High

performer

Very high

performer

| Sales Performance | 1 | 2 | 3 | 4 | 5 | 6 | 7 |
| --- | --- | --- | --- | --- | --- | --- | --- |
| Customer Retention | 1 | 2 | 3 | 4 | 5 | 6 | 7 |
| Customer Satisfaction | 1 | 2 | 3 | 4 | 5 | 6 | 7 |
| Ability to manage and coach employees | 1 | 2 | 3 | 4 | 5 | 6 | 7 |

Please rate how **important** each of these factors is for Taylor Devani’s job performance at DeltaCom (1=not important to 7=very important):

Not

important

Somewhat

important

Important

Very

important

| Sales Performance | 1 | 2 | 3 | 4 | 5 | 6 | 7 |
| --- | --- | --- | --- | --- | --- | --- | --- |
| Customer Retention | 1 | 2 | 3 | 4 | 5 | 6 | 7 |
| Customer Satisfaction | 1 | 2 | 3 | 4 | 5 | 6 | 7 |
| Ability to manage and coach employees | 1 | 2 | 3 | 4 | 5 | 6 | 7 |

Please continue to the next page…

Please give your opinion about the **causes of Taylor Devani’s successes** by assigning a percentage to each of the following four causes, such that the four causes together **sum to 100%**.

% due to Taylor’s abilities and personality

| □  0 | □  5 | □  10 | □  15 | □  20 | □  25 | □  30 | □  35 | □  40 | □  45 | □  50 | □  55 | □  60 | □  65 | □  70 | □  75 | □  80 | □  85 | □  90 | □  95 | □  100 |
| --- | --- | --- | --- | --- | --- | --- | --- | --- | --- | --- | --- | --- | --- | --- | --- | --- | --- | --- | --- | --- |

% due to the amount of effort and attention Taylor applied

| □  0 | □  5 | □  10 | □  15 | □  20 | □  25 | □  30 | □  35 | □  40 | □  45 | □  50 | □  55 | □  60 | □  65 | □  70 | □  75 | □  80 | □  85 | □  90 | □  95 | □  100 |
| --- | --- | --- | --- | --- | --- | --- | --- | --- | --- | --- | --- | --- | --- | --- | --- | --- | --- | --- | --- | --- |

% due to Taylor’s job responsibilities, DeltaCom’s expectations, and the resources provided

| □  0 | □  5 | □  10 | □  15 | □  20 | □  25 | □  30 | □  35 | □  40 | □  45 | □  50 | □  55 | □  60 | □  65 | □  70 | □  75 | □  80 | □  85 | □  90 | □  95 | □  100 |
| --- | --- | --- | --- | --- | --- | --- | --- | --- | --- | --- | --- | --- | --- | --- | --- | --- | --- | --- | --- | --- |

% due to chance and random luck

| □  0 | □  5 | □  10 | □  15 | □  20 | □  25 | □  30 | □  35 | □  40 | □  45 | □  50 | □  55 | □  60 | □  65 | □  70 | □  75 | □  80 | □  85 | □  90 | □  95 | □  100 |
| --- | --- | --- | --- | --- | --- | --- | --- | --- | --- | --- | --- | --- | --- | --- | --- | --- | --- | --- | --- | --- |

**PLEASE CHECK: Do the above four numbers add to 100%? If not, please revise.**

Please give your opinion about the **causes of Taylor Devani’s failures** by assigning a percentage to each of the following four causes, such that the four causes together **sum to 100%**.

% due to Taylor’s abilities and personality

| □  0 | □  5 | □  10 | □  15 | □  20 | □  25 | □  30 | □  35 | □  40 | □  45 | □  50 | □  55 | □  60 | □  65 | □  70 | □  75 | □  80 | □  85 | □  90 | □  95 | □  100 |
| --- | --- | --- | --- | --- | --- | --- | --- | --- | --- | --- | --- | --- | --- | --- | --- | --- | --- | --- | --- | --- |

% due to the amount of effort and attention Taylor applied

| □  0 | □  5 | □  10 | □  15 | □  20 | □  25 | □  30 | □  35 | □  40 | □  45 | □  50 | □  55 | □  60 | □  65 | □  70 | □  75 | □  80 | □  85 | □  90 | □  95 | □  100 |
| --- | --- | --- | --- | --- | --- | --- | --- | --- | --- | --- | --- | --- | --- | --- | --- | --- | --- | --- | --- | --- |

% due to Taylor’s job responsibilities, DeltaCom’s expectations, and the resources provided

| □  0 | □  5 | □  10 | □  15 | □  20 | □  25 | □  30 | □  35 | □  40 | □  45 | □  50 | □  55 | □  60 | □  65 | □  70 | □  75 | □  80 | □  85 | □  90 | □  95 | □  100 |
| --- | --- | --- | --- | --- | --- | --- | --- | --- | --- | --- | --- | --- | --- | --- | --- | --- | --- | --- | --- | --- |

% due to chance and random luck

| □  0 | □  5 | □  10 | □  15 | □  20 | □  25 | □  30 | □  35 | □  40 | □  45 | □  50 | □  55 | □  60 | □  65 | □  70 | □  75 | □  80 | □  85 | □  90 | □  95 | □  100 |
| --- | --- | --- | --- | --- | --- | --- | --- | --- | --- | --- | --- | --- | --- | --- | --- | --- | --- | --- | --- | --- |

**PLEASE CHECK: Do the above four numbers add to 100%? If not, please revise.**

Please continue to the next page…

Please print your name: _________________

The name of the person playing District Manager, Taylor Devani: _________________

Please tear off the blue pages and place them in the envelope provided. When you are ready, you may begin your 20-minute meeting with Taylor to discuss performance issues. After the meeting, you will complete a questionnaire labeled Part 2. Please do NOT complete Part 2 until ***after*** the 20-minute meeting. Please stay in role during the entire exercise.
