# Supplementary material for: The future of feedback: Motivating performance improvement through future-focused feedback
Source: PLoS One. 2020 Jun 19;15(6):e0234444. doi: 10.1371/journal.pone.0234444 (PMC7304587; doi:10.1371/journal.pone.0234444)
Supplement: S6 Text — (DOCX) [file pone.0234444.s006.docx]

**The future of feedback: Motivating performance improvement**

Jackie Gnepp, Joshua Klayman, Ian O. Williamson, Sema Barlas

**S7 Text. Study 2 post-discussion questionnaire – District Manager, pre-post group.**

**DELTACOM CORPORATION EXERCISE**

**Please print your name here:**

**Please answer *every* question asked below (regardless of the role you played). This survey may include items you answered previously. Please do not look back at Part 1; rather, give your current thinking on these items. Please stay in role while completing this questionnaire and answer the questions as District Manager, Taylor Devani.**

Please rate the **content** of the feedback that Chris Sinopoli gave to Taylor Devani from **0 = almost all negative** to **10 = almost all positive**.

Almost all negative Equal Almost all positive

| □  0 | □  1 | □  2 | □  3 | □  4 | □  5 | □  6 | □  7 | □  8 | □  9 | □  10 |
| --- | --- | --- | --- | --- | --- | --- | --- | --- | --- | --- |

Please rate the **accuracy** of the feedback that Chris Sinopoli gave to Taylor Devani from **0%** to **100% accurate**.

| □  0 | □  5 | □  10 | □  15 | □  20 | □  25 | □  30 | □  35 | □  40 | □  45 | □  50 | □  55 | □  60 | □  65 | □  70 | □  75 | □  80 | □  85 | □  90 | □  95 | □  100 |
| --- | --- | --- | --- | --- | --- | --- | --- | --- | --- | --- | --- | --- | --- | --- | --- | --- | --- | --- | --- | --- |

Please rate how **qualified** Chris Sinopoli was to give feedback to Taylor Devani from **0 = unqualified** to **10 = completely qualified**.

| □  0 | □  1 | □  2 | □  3 | □  4 | □  5 | □  6 | □  7 | □  8 | □  9 | □  10 |
| --- | --- | --- | --- | --- | --- | --- | --- | --- | --- | --- |

Please continue to the next page…Please give your opinion about the **causes of Taylor Devani’s successes** by assigning a percentage to each of the following four causes, such that the four causes together **sum to 100%**.

% due to Taylor’s abilities and personality

| □  0 | □  5 | □  10 | □  15 | □  20 | □  25 | □  30 | □  35 | □  40 | □  45 | □  50 | □  55 | □  60 | □  65 | □  70 | □  75 | □  80 | □  85 | □  90 | □  95 | □  100 |
| --- | --- | --- | --- | --- | --- | --- | --- | --- | --- | --- | --- | --- | --- | --- | --- | --- | --- | --- | --- | --- |

% due to the amount of effort and attention Taylor applied

| □  0 | □  5 | □  10 | □  15 | □  20 | □  25 | □  30 | □  35 | □  40 | □  45 | □  50 | □  55 | □  60 | □  65 | □  70 | □  75 | □  80 | □  85 | □  90 | □  95 | □  100 |
| --- | --- | --- | --- | --- | --- | --- | --- | --- | --- | --- | --- | --- | --- | --- | --- | --- | --- | --- | --- | --- |

% due to Taylor’s job responsibilities, DeltaCom’s expectations, and the resources provided

| □  0 | □  5 | □  10 | □  15 | □  20 | □  25 | □  30 | □  35 | □  40 | □  45 | □  50 | □  55 | □  60 | □  65 | □  70 | □  75 | □  80 | □  85 | □  90 | □  95 | □  100 |
| --- | --- | --- | --- | --- | --- | --- | --- | --- | --- | --- | --- | --- | --- | --- | --- | --- | --- | --- | --- | --- |

% due to chance and random luck

| □  0 | □  5 | □  10 | □  15 | □  20 | □  25 | □  30 | □  35 | □  40 | □  45 | □  50 | □  55 | □  60 | □  65 | □  70 | □  75 | □  80 | □  85 | □  90 | □  95 | □  100 |
| --- | --- | --- | --- | --- | --- | --- | --- | --- | --- | --- | --- | --- | --- | --- | --- | --- | --- | --- | --- | --- |

**PLEASE CHECK: Do the above four numbers add to 100%? If not, please revise.**

Please give your opinion about the **causes of Taylor Devani’s failures** by assigning a percentage to each of the following four causes, such that the four causes together **sum to 100%**.

% due to Taylor’s abilities and personality

| □  0 | □  5 | □  10 | □  15 | □  20 | □  25 | □  30 | □  35 | □  40 | □  45 | □  50 | □  55 | □  60 | □  65 | □  70 | □  75 | □  80 | □  85 | □  90 | □  95 | □  100 |
| --- | --- | --- | --- | --- | --- | --- | --- | --- | --- | --- | --- | --- | --- | --- | --- | --- | --- | --- | --- | --- |

% due to the amount of effort and attention Taylor applied

| □  0 | □  5 | □  10 | □  15 | □  20 | □  25 | □  30 | □  35 | □  40 | □  45 | □  50 | □  55 | □  60 | □  65 | □  70 | □  75 | □  80 | □  85 | □  90 | □  95 | □  100 |
| --- | --- | --- | --- | --- | --- | --- | --- | --- | --- | --- | --- | --- | --- | --- | --- | --- | --- | --- | --- | --- |

% due to Taylor’s job responsibilities, DeltaCom’s expectations, and the resources provided

| □  0 | □  5 | □  10 | □  15 | □  20 | □  25 | □  30 | □  35 | □  40 | □  45 | □  50 | □  55 | □  60 | □  65 | □  70 | □  75 | □  80 | □  85 | □  90 | □  95 | □  100 |
| --- | --- | --- | --- | --- | --- | --- | --- | --- | --- | --- | --- | --- | --- | --- | --- | --- | --- | --- | --- | --- |

% due to chance and random luck

| □  0 | □  5 | □  10 | □  15 | □  20 | □  25 | □  30 | □  35 | □  40 | □  45 | □  50 | □  55 | □  60 | □  65 | □  70 | □  75 | □  80 | □  85 | □  90 | □  95 | □  100 |
| --- | --- | --- | --- | --- | --- | --- | --- | --- | --- | --- | --- | --- | --- | --- | --- | --- | --- | --- | --- | --- |

**PLEASE CHECK: Do the above four numbers add to 100%? If not, please revise.**

## Please continue to the next page…Please indicate the extent to which you agree with the following statements about the Regional Manager Chris Sinopoli’s behavior during the session. (1= strongly disagree to 7 = strongly agree)

**During the feedback session the Regional Manager….**

Strongly

disagree

Slightly

disagree

Slightly

agree

Strongly

agree

| Invited the District Manger to suggest ways to improve upon past performance and outcomes | 1 | 2 | 3 | 4 | 5 | 6 | 7 |
| --- | --- | --- | --- | --- | --- | --- | --- |
| Offered to provide any assistance the District Manager needed in order to respond to his/her feedback | 1 | 2 | 3 | 4 | 5 | 6 | 7 |
| Said that his/her feedback was consistent with prior precedent and established practice in the company | 1 | 2 | 3 | 4 | 5 | 6 | 7 |
| Offered to do something for the District Manager in the future in return for carrying out his/her requests | 1 | 2 | 3 | 4 | 5 | 6 | 7 |
| Provided information or evidence to show why his/her feedback would likely to lead to success | 1 | 2 | 3 | 4 | 5 | 6 | 7 |
| Described a clear, inspiring vision of what following his/her feedback could accomplish | 1 | 2 | 3 | 4 | 5 | 6 | 7 |
| Used threats or warnings when trying to get the District Manager to accept their feedback | 1 | 2 | 3 | 4 | 5 | 6 | 7 |
| Said that following his\her feedback could provide an opportunity to do something really exciting and worthwhile | 1 | 2 | 3 | 4 | 5 | 6 | 7 |
| Talked about values and ideals when presenting his/her feedback | 1 | 2 | 3 | 4 | 5 | 6 | 7 |
| Explained how following his/her feedback could help the District Manager's career | 1 | 2 | 3 | 4 | 5 | 6 | 7 |
| Offered to do something for the District Manager in exchange for following his/her requests | 1 | 2 | 3 | 4 | 5 | 6 | 7 |
| Explained why accepting his/her feedback would be good for the District Manager | 1 | 2 | 3 | 4 | 5 | 6 | 7 |
| Demanded that the District Manager carry out a request | 1 | 2 | 3 | 4 | 5 | 6 | 7 |
| Couched his/her feedback as being consistent with official company strategy and policy | 1 | 2 | 3 | 4 | 5 | 6 | 7 |
| Asked the District Manager to suggest things he/she could do to achieve a task objective or resolve a problem | 1 | 2 | 3 | 4 | 5 | 6 | 7 |
| Praised the District Manager's skill or knowledge | 1 | 2 | 3 | 4 | 5 | 6 | 7 |
| Described the benefits the District Manager could gain from following his/her feedback | 1 | 2 | 3 | 4 | 5 | 6 | 7 |
| Offered to provide resources the District Manager would need to respond to his/her feedback | 1 | 2 | 3 | 4 | 5 | 6 | 7 |
| Used facts and logic to make a persuasive case for his/her point of view | 1 | 2 | 3 | 4 | 5 | 6 | 7 |
| Provided praise for the District Manager's past performance or achievements | 1 | 2 | 3 | 4 | 5 | 6 | 7 |

Please continue to the next page…

**Please answer the following questions about the District Manager Taylor Devani**

Please rate Taylor Devani’s **level of job performance** on each of the following factors (1=very low performer to 7=very high performer):

Very low

performer

Low

performer

High

performer

Very high

performer

| Sales Performance | 1 | 2 | 3 | 4 | 5 | 6 | 7 |
| --- | --- | --- | --- | --- | --- | --- | --- |
| Customer Retention | 1 | 2 | 3 | 4 | 5 | 6 | 7 |
| Customer Satisfaction | 1 | 2 | 3 | 4 | 5 | 6 | 7 |
| Ability to manage and coach employees | 1 | 2 | 3 | 4 | 5 | 6 | 7 |

Please rate how **important** each of these factors is for Taylor Devani’s job performance at DeltaCom (1=not important to 7=very important):

Not

important

Somewhat

important

Important

Very

important

| Sales Performance | 1 | 2 | 3 | 4 | 5 | 6 | 7 |
| --- | --- | --- | --- | --- | --- | --- | --- |
| Customer Retention | 1 | 2 | 3 | 4 | 5 | 6 | 7 |
| Customer Satisfaction | 1 | 2 | 3 | 4 | 5 | 6 | 7 |
| Ability to manage and coach employees | 1 | 2 | 3 | 4 | 5 | 6 | 7 |

## Please indicate the extent to which you agree with the following statements about the District Manager Taylor Devani’s behavior during the exercise. (1= strongly disagree to 7 = strongly agree)

**During the feedback session the District Manager….**

Strongly

disagree

Slightly

disagree

Slightly

agree

Strongly

agree

| Made excuses or blamed others for his/her poor performance | 1 | 2 | 3 | 4 | 5 | 6 | 7 |
| --- | --- | --- | --- | --- | --- | --- | --- |
| Was accountable for his/her performance | 1 | 2 | 3 | 4 | 5 | 6 | 7 |
| Was defensive during the meeting | 1 | 2 | 3 | 4 | 5 | 6 | 7 |
| Was receptive to the feedback from the Regional Manager | 1 | 2 | 3 | 4 | 5 | 6 | 7 |
| Acknowledge valid points made by the Regional Manager | 1 | 2 | 3 | 4 | 5 | 6 | 7 |

Please continue to the next page…

## Please indicate the extent to which you agree with the following statements about the feedback session (1= strongly disagree to 7 = strongly agree). Please respond to the items in your role as District Manager, Taylor Devani:

Strongly

disagree

Slightly

disagree

Slightly

agree

Strongly

agree

| The feedback discussion focused mostly on your future behavior. | 1 | 2 | 3 | 4 | 5 | 6 | 7 |
| --- | --- | --- | --- | --- | --- | --- | --- |
| You and Chris came to agreement. | 1 | 2 | 3 | 4 | 5 | 6 | 7 |
| The feedback you received was appropriate for the work you have completed. | 1 | 2 | 3 | 4 | 5 | 6 | 7 |
| Chris treated you in a polite manner. | 1 | 2 | 3 | 4 | 5 | 6 | 7 |
| Based on the feedback, you are now motivated to change your behavior. | 1 | 2 | 3 | 4 | 5 | 6 | 7 |
| At the end of the meeting, you and Chris had similar views. | 1 | 2 | 3 | 4 | 5 | 6 | 7 |
| The feedback was justified, given your performance. | 1 | 2 | 3 | 4 | 5 | 6 | 7 |
| Chris refrained from improper remarks or comments. | 1 | 2 | 3 | 4 | 5 | 6 | 7 |
| You and Chris spent a large part of this session generating new ideas for your next steps. | 1 | 2 | 3 | 4 | 5 | 6 | 7 |
| You see the value of acting on Chris’s suggestions. | 1 | 2 | 3 | 4 | 5 | 6 | 7 |
| Chris treated you with respect. | 1 | 2 | 3 | 4 | 5 | 6 | 7 |
| The feedback Chris gave you reflected what you have contributed to DeltaCom. | 1 | 2 | 3 | 4 | 5 | 6 | 7 |
| Chris will probably recommend you for promotion based on the feedback session. | 1 | 2 | 3 | 4 | 5 | 6 | 7 |
| You and Chris now share the same opinion about what you need to do to be successful. | 1 | 2 | 3 | 4 | 5 | 6 | 7 |
| Chris treated you with dignity. | 1 | 2 | 3 | 4 | 5 | 6 | 7 |
| You will likely change your behavior, based on the feedback received. | 1 | 2 | 3 | 4 | 5 | 6 | 7 |
| Chris’s feedback to you accurately reflected the effort you have put into your work. | 1 | 2 | 3 | 4 | 5 | 6 | 7 |
| The feedback conversation centered on what will make you most successful going forward. | 1 | 2 | 3 | 4 | 5 | 6 | 7 |

Please continue to the next page…

Thank you for completing this questionnaire. Now please tell us about yourself:

Are you Male or Female? (Circle one)

What is your age? ___________________

What is the highest level of education you have completed? _______________________

What is the nature of your current employment or most recent full-time job? Circle the one that fits best:

○ Front-line employee ○ Self-employed individual

○ Professional practice ○ Entrepreneur/small business

○ Junior management ○ Middle management ○ Executive/upper management

What do you think of as your main cultural identity? Please include any national and/or ethnic descriptions that characterize how you think of yourself—you may use one (for example, “Chinese” or “Welsh”) or more than one (e.g., “Malaysian-Indian”), as you wish:

_________________________________________

Thank you. Please hand in this packet.
