# Supplementary material for: The future of feedback: Motivating performance improvement through future-focused feedback
Source: PLoS One. 2020 Jun 19;15(6):e0234444. doi: 10.1371/journal.pone.0234444 (PMC7304587; doi:10.1371/journal.pone.0234444)
Supplement: S7 Text — (DOCX) [file pone.0234444.s007.docx]

**The future of feedback: Motivating performance improvement**

Jackie Gnepp, Joshua Klayman, Ian O. Williamson, Sema Barlas

**S8 Text. Study 2 post-discussion questionnaire – both roles, post-only group.**

**DELTACOM CORPORATION EXERCISE**

**Please write in the names of the people playing each role in this exercise. Then, circle your own name and the role you played.**

Regional Manager (Chris Sinopoli):

District Manager (Taylor Devani):

**Please answer *every* question asked below (regardless of the role you played). If you were the Regional Manager Chris Sinopoli please answer about yourself. If you were the District Manager Taylor Devani please answer about yourself.**

Please rate the **content** of the feedback that Chris Sinopoli gave to Taylor Devani from **0 = almost all negative** to **10 = almost all positive**.

Almost all negative Equal Almost all positive

| □  0 | □  1 | □  2 | □  3 | □  4 | □  5 | □  6 | □  7 | □  8 | □  9 | □  10 |
| --- | --- | --- | --- | --- | --- | --- | --- | --- | --- | --- |

Please rate the **accuracy** of the feedback that Chris Sinopoli gave to Taylor Devani from **0%** to **100% accurate**.

| □  0 | □  5 | □  10 | □  15 | □  20 | □  25 | □  30 | □  35 | □  40 | □  45 | □  50 | □  55 | □  60 | □  65 | □  70 | □  75 | □  80 | □  85 | □  90 | □  95 | □  100 |
| --- | --- | --- | --- | --- | --- | --- | --- | --- | --- | --- | --- | --- | --- | --- | --- | --- | --- | --- | --- | --- |

Please rate how **qualified** Chris Sinopoli was to give feedback to Taylor Devani from **0 = unqualified** to **10 = completely qualified**.

| □  0 | □  1 | □  2 | □  3 | □  4 | □  5 | □  6 | □  7 | □  8 | □  9 | □  10 |
| --- | --- | --- | --- | --- | --- | --- | --- | --- | --- | --- |

Please continue to the next page…Please give your opinion about the **causes of Taylor Devani’s successes** by assigning a percentage to each of the following four causes, such that the four causes together **sum to 100%**.

% due to Taylor’s abilities and personality

| □  0 | □  5 | □  10 | □  15 | □  20 | □  25 | □  30 | □  35 | □  40 | □  45 | □  50 | □  55 | □  60 | □  65 | □  70 | □  75 | □  80 | □  85 | □  90 | □  95 | □  **100** |
| --- | --- | --- | --- | --- | --- | --- | --- | --- | --- | --- | --- | --- | --- | --- | --- | --- | --- | --- | --- | --- |

% due to the amount of effort and attention Taylor applied

| □  0 | □  5 | □  10 | □  15 | □  20 | □  25 | □  30 | □  35 | □  40 | □  45 | □  50 | □  55 | □  60 | □  65 | □  70 | □  75 | □  80 | □  85 | □  90 | □  95 | □  **100** |
| --- | --- | --- | --- | --- | --- | --- | --- | --- | --- | --- | --- | --- | --- | --- | --- | --- | --- | --- | --- | --- |

% due to Taylor’s job responsibilities, DeltaCom’s expectations, and the resources provided

| □  0 | □  5 | □  10 | □  15 | □  20 | □  25 | □  30 | □  35 | □  40 | □  45 | □  50 | □  55 | □  60 | □  65 | □  70 | □  75 | □  80 | □  85 | □  90 | □  95 | □  **100** |
| --- | --- | --- | --- | --- | --- | --- | --- | --- | --- | --- | --- | --- | --- | --- | --- | --- | --- | --- | --- | --- |

% due to chance and random luck

| □  0 | □  5 | □  10 | □  15 | □  20 | □  25 | □  30 | □  35 | □  40 | □  45 | □  50 | □  55 | □  60 | □  65 | □  70 | □  75 | □  80 | □  85 | □  90 | □  95 | □  **100** |
| --- | --- | --- | --- | --- | --- | --- | --- | --- | --- | --- | --- | --- | --- | --- | --- | --- | --- | --- | --- | --- |

**PLEASE CHECK: Do the above four numbers add to 100%? If not, please revise.**

Please give your opinion about the **causes of Taylor Devani’s failures** by assigning a percentage to each of the following four causes, such that the four causes together **sum to 100%**.

% due to Taylor’s abilities and personality

| □  0 | □  5 | □  10 | □  15 | □  20 | □  25 | □  30 | □  35 | □  40 | □  45 | □  50 | □  55 | □  60 | □  65 | □  70 | □  75 | □  80 | □  85 | □  90 | □  95 | □  **100** |
| --- | --- | --- | --- | --- | --- | --- | --- | --- | --- | --- | --- | --- | --- | --- | --- | --- | --- | --- | --- | --- |

% due to the amount of effort and attention Taylor applied

| □  0 | □  5 | □  10 | □  15 | □  20 | □  25 | □  30 | □  35 | □  40 | □  45 | □  50 | □  55 | □  60 | □  65 | □  70 | □  75 | □  80 | □  85 | □  90 | □  95 | □  **100** |
| --- | --- | --- | --- | --- | --- | --- | --- | --- | --- | --- | --- | --- | --- | --- | --- | --- | --- | --- | --- | --- |

% due to Taylor’s job responsibilities, DeltaCom’s expectations, and the resources provided

| □  0 | □  5 | □  10 | □  15 | □  20 | □  25 | □  30 | □  35 | □  40 | □  45 | □  50 | □  55 | □  60 | □  65 | □  70 | □  75 | □  80 | □  85 | □  90 | □  95 | □  **100** |
| --- | --- | --- | --- | --- | --- | --- | --- | --- | --- | --- | --- | --- | --- | --- | --- | --- | --- | --- | --- | --- |

% due to chance and random luck

| □  0 | □  5 | □  10 | □  15 | □  20 | □  25 | □  30 | □  35 | □  40 | □  45 | □  50 | □  55 | □  60 | □  65 | □  70 | □  75 | □  80 | □  85 | □  90 | □  95 | □  **100** |
| --- | --- | --- | --- | --- | --- | --- | --- | --- | --- | --- | --- | --- | --- | --- | --- | --- | --- | --- | --- | --- |

**PLEASE CHECK: Do the above four numbers add to 100%? If not, please revise.**

Please continue to the next page…

**Please answer the following questions about the District Manager Taylor Devani**

Please rate Taylor Devani’s **level of job performance** on each of the following factors (1=very low performer to 7=very high performer):

| Very Low Performer | Low Performer | High Performer | Very High Performer |
| --- | --- | --- | --- |

| Sales Performance | 1 | 2 | 3 | 4 | 5 | 6 | 7 |
| --- | --- | --- | --- | --- | --- | --- | --- |
| Customer Retention | 1 | 2 | 3 | 4 | 5 | 6 | 7 |
| Customer Satisfaction | 1 | 2 | 3 | 4 | 5 | 6 | 7 |
| Ability to manage and coach employees | 1 | 2 | 3 | 4 | 5 | 6 | 7 |

Please rate how **important** each of these factors is for Taylor Devani’s job performance at DeltaCom (1=not important to 7=very important):

| Not Important | Somewhat Important | Important | Very Important |
| --- | --- | --- | --- |

| Sales Performance | 1 | 2 | 3 | 4 | 5 | 6 | 7 |
| --- | --- | --- | --- | --- | --- | --- | --- |
| Customer Retention | 1 | 2 | 3 | 4 | 5 | 6 | 7 |
| Customer Satisfaction | 1 | 2 | 3 | 4 | 5 | 6 | 7 |
| Ability to manage and coach employees | 1 | 2 | 3 | 4 | 5 | 6 | 7 |

## Please indicate the extent to which you agree with the following statements about the District Manager Taylor Devani’s behavior during the exercise. (1= strongly disagree to 7 = strongly agree)

| Strongly  Disagree | Slightly  Disagree | Slightly  Agree | Strongly  Agree |
| --- | --- | --- | --- |

**During the exercise the District Manager….**

| Was rude | 1 | 2 | 3 | 4 | 5 | 6 | 7 |
| --- | --- | --- | --- | --- | --- | --- | --- |
| Made excuses or blamed others for his/her poor performance | 1 | 2 | 3 | 4 | 5 | 6 | 7 |
| Was effective at expressing ideas and opinions | 1 | 2 | 3 | 4 | 5 | 6 | 7 |
| Showed disrespect towards me. | 1 | 2 | 3 | 4 | 5 | 6 | 7 |
| Was accountable for his/her performance | 1 | 2 | 3 | 4 | 5 | 6 | 7 |
| Was tactul | 1 | 2 | 3 | 4 | 5 | 6 | 7 |
| Was defensive during the meeting | 1 | 2 | 3 | 4 | 5 | 6 | 7 |
| Was receptive to the feedback from the Regional Manager | 1 | 2 | 3 | 4 | 5 | 6 | 7 |
| Was insensitive | 1 | 2 | 3 | 4 | 5 | 6 | 7 |
| Acknowledge vaild points made by the Regional Manager | 1 | 2 | 3 | 4 | 5 | 6 | 7 |
| Was effective at listening to ideas and opinions | 1 | 2 | 3 | 4 | 5 | 6 | 7 |
| Was polite. | 1 | 2 | 3 | 4 | 5 | 6 | 7 |

Please continue to the next page…

## Please indicate the extent to which you agree with the following statements about the Regional Manager Chris Sinopoli’s behavior during the exercise. (1= strongly disagree to 7 = strongly agree)

| Strongly  Disagree | Slightly  Disagree | Slightly  Agree | Strongly  Agree |
| --- | --- | --- | --- |

**During the exercise the Regional Manager….**

| Invited the District Manger to suggest ways to improve upon past performance and outcomes | 1 | 2 | 3 | 4 | 5 | 6 | 7 |
| --- | --- | --- | --- | --- | --- | --- | --- |
| Offered to provide any assistance the District Manager needed in order to respond to his/her feedback | 1 | 2 | 3 | 4 | 5 | 6 | 7 |
| Said that his/her feedback was consistent with prior precedent and established practice in the company | 1 | 2 | 3 | 4 | 5 | 6 | 7 |
| Was effective at listening to ideas and opinions | 1 | 2 | 3 | 4 | 5 | 6 | 7 |
| Offered to do something for the District Manager in the future in return for carrying out his/her requests | 1 | 2 | 3 | 4 | 5 | 6 | 7 |
| Provided information or evidence to show why his/her feedback would likely to lead to success | 1 | 2 | 3 | 4 | 5 | 6 | 7 |
| Was tactful | 1 | 2 | 3 | 4 | 5 | 6 | 7 |
| Was very effective at delivering feedback | 1 | 2 | 3 | 4 | 5 | 6 | 7 |
| Described a clear, inspiring vision of what following his/her feedback could accomplish | 1 | 2 | 3 | 4 | 5 | 6 | 7 |
| Used threats or warnings when trying to get the District Manager to accept their feedback | 1 | 2 | 3 | 4 | 5 | 6 | 7 |
| Was rude | 1 | 2 | 3 | 4 | 5 | 6 | 7 |
| Said that following his\her feedback could provide an opportunity to do something really exciting and worthwhile | 1 | 2 | 3 | 4 | 5 | 6 | 7 |
| Talked about values and ideals when presenting his/her feedback | 1 | 2 | 3 | 4 | 5 | 6 | 7 |
| Explained how following his/her feedback could help the District Manager's career | 1 | 2 | 3 | 4 | 5 | 6 | 7 |
| Offered to do something for the District Manager in exchange for following his/her requests | 1 | 2 | 3 | 4 | 5 | 6 | 7 |
| Was effective at expressing ideas and opinions | 1 | 2 | 3 | 4 | 5 | 6 | 7 |
| Explained why accepting his/her feedback would be good for the District Manager | 1 | 2 | 3 | 4 | 5 | 6 | 7 |
| Demanded that the District Manager carry out a request | 1 | 2 | 3 | 4 | 5 | 6 | 7 |
| Showed disrespect towards the District Manager | 1 | 2 | 3 | 4 | 5 | 6 | 7 |
| Couched his/her feedback as being consistent with official company strategy and policy | 1 | 2 | 3 | 4 | 5 | 6 | 7 |
| Asked the District Manager to suggest things he/she could do to achieve a task objective or resolve a problem | 1 | 2 | 3 | 4 | 5 | 6 | 7 |
| Praised the District Manager's skill or knowledge | 1 | 2 | 3 | 4 | 5 | 6 | 7 |
| Was very effective at conveying feedback during the meeting | 1 | 2 | 3 | 4 | 5 | 6 | 7 |
| Described the benefits the District Manager could gain from following his/her feedback | 1 | 2 | 3 | 4 | 5 | 6 | 7 |
| Was insensitive | 1 | 2 | 3 | 4 | 5 | 6 | 7 |
| Offered to provide resources the District Manager would need to respond to his/her feedback | 1 | 2 | 3 | 4 | 5 | 6 | 7 |
| Was polite. | 1 | 2 | 3 | 4 | 5 | 6 | 7 |
| Used facts and logic to make a persuasive case for his/her point of view | 1 | 2 | 3 | 4 | 5 | 6 | 7 |
| Provided praise for the District Manager's past performance or achievements | 1 | 2 | 3 | 4 | 5 | 6 | 7 |

Thank you for completing this questionnaire. Now please tell us about yourself:

Are you Male or Female? (Circle one)

What is your age? ___________________

What is the highest level of education you have completed? _______________________

What is the nature of your current employment or most recent full-time job? Circle the one that fits best:

○ Front-line employee ○ Self-employed individual

○ Professional practice ○ Entrepreneur/small business

○ Junior management ○ Middle management ○ Executive/upper management

What do you think of as your main cultural identity? Please include any national and/or ethnic descriptions that characterize how you think of yourself—you may use one (for example, “Chinese” or “Welsh”) or more than one (e.g., “Malaysian-Indian”), as you wish:

_________________________________________

Thank you. Please hand in this packet.
