# Supplementary material for: The future of feedback: Motivating performance improvement through future-focused feedback
Source: PLoS One. 2020 Jun 19;15(6):e0234444. doi: 10.1371/journal.pone.0234444 (PMC7304587; doi:10.1371/journal.pone.0234444)
Supplement: S8 Text — (DOCX) [file pone.0234444.s008.docx]

**The future of feedback: Motivating performance improvement**

**through future-focused feedback**

Jackie Gnepp, Joshua Klayman, Ian O. Williamson, Sema Barlas

**S12 Text. Study 3 guidelines added to instructions – two versions.**

A. Performance version

**DeltaCom Guidelines for Giving Feedback**

We at DeltaCom believe that performance feedback is really important for improving performance. In an effort to increase the effectiveness of the feedback we provide, DeltaCom has instituted new guidelines based on the latest in management thinking. You are asked to keep these guidelines in mind when giving feedback.

Feedback at DeltaCom is focused on the person’s performance. While it is important for you to acknowledge strengths and weaknesses, DeltaCom asks that you focus the feedback conversation on how the person has performed in their job.

1. Establish early on that your intentions are to help the person think about their performance so that they understand how they could have done better in their job.

2. DeltaCom holds high standards. As appropriate, discuss our values, ideals, and performance expectations so that the person knows how their performance compares to expectations.

3. Review the person’s past performance, providing praise where warranted. Where performance is poor, illustrate it with facts: Feedback is only useful to the extent that it is understood and accepted by the person receiving it. Remember, the goal is quality feedback.

4. DeltaCom employees are motivated to succeed. Let the person know you have every confidence in their ability to honestly evaluate their job performance.

5. Initiate a discussion of their performance. What patterns does the person see in their performance? How well does their performance meet DeltaCom’s high standards? How could DeltaCom have helped? What are reasonable targets for their performance?

6. Put your heads together to review their performance. Make sure the person receiving feedback leaves the session knowing what they have done well and where they need to improve.

B. Improvement version

**DeltaCom Guidelines for Giving Feedback**

We at DeltaCom believe that performance feedback is really important for improving performance. In an effort to increase the effectiveness of the feedback we provide, DeltaCom has instituted new guidelines based on the latest in management thinking. You are asked to keep these guidelines in mind when giving feedback.

Feedback at DeltaCom is focused on future performance. While it is important for you to acknowledge strengths and weaknesses, DeltaCom asks that you focus the feedback conversation on next steps and future actions.

1. Establish early on that your intentions are to help the person think about their future performance, so that they understand how they can do better in their current job or the next.

2. DeltaCom holds high standards. As appropriate, discuss our values, ideals, and performance expectations so that the person knows what will be expected of them in the future.

3. Review the person’s past performance, providing praise where warranted. Where performance is poor, stick to the facts: Do not get sucked into a discussion of how or why the past happened, or whether it was justified. Remember, the goal is a better future.

4. DeltaCom employees are motivated to succeed. Let the person know you have every confidence in their ability to do better in the future.

5. Initiate a discussion of future plans and milestones. What ideas does the person have for how to strengthen their future performance? How can they meet DeltaCom’s high standards in the future? How can DeltaCom help? What will be reasonable targets for the short- and medium-term?

6. Put your heads together to develop solutions for the future. Make sure the person receiving feedback leaves the session knowing what steps and actions to take to perform better in the future.
