# Supplementary material for: The future of feedback: Motivating performance improvement through future-focused feedback
Source: PLoS One. 2020 Jun 19;15(6):e0234444. doi: 10.1371/journal.pone.0234444 (PMC7304587; doi:10.1371/journal.pone.0234444)
Supplement: S9 Text — (DOCX) [file pone.0234444.s009.docx]

**The future of feedback: Motivating performance improvement**

**through future-focused feedback**

Jackie Gnepp, Joshua Klayman, Ian O. Williamson, Sema Barlas

**S13 Text. Study 3 post-discussion questionnaire – Regional Manager.**

**DELTACOM CORPORATION EXERCISE**

**Please answer *every* question asked below. Please continue in your role as Chris Sinopoli, and provide Chris’s answers to these questions.**

Please rate the **content** of the feedback that you gave to Taylor Devani

from **0 = almost all negative** to **10 = almost all positive**.

Almost all negative Equal Almost all positive

| □  0 | □  1 | □  2 | □  3 | □  4 | □  5 | □  6 | □  7 | □  8 | □  9 | □  10 |
| --- | --- | --- | --- | --- | --- | --- | --- | --- | --- | --- |

Please rate the accuracy of the feedback that you gave to Taylor Devani

from **0%** to **100% accurate**.

| □  0 | □  5 | □  10 | □  15 | □  20 | □  25 | □  30 | □  35 | □  40 | □  45 | □  50 | □  55 | □  60 | □  65 | □  70 | □  75 | □  80 | □  85 | □  90 | □  95 | □  100 |
| --- | --- | --- | --- | --- | --- | --- | --- | --- | --- | --- | --- | --- | --- | --- | --- | --- | --- | --- | --- | --- |

Please rate how qualified you were to give feedback to Taylor Devani

from **0 = unqualified** to **10 = completely qualified**.

| □  0 | □  1 | □  2 | □  3 | □  4 | □  5 | □  6 | □  7 | □  8 | □  9 | □  10 |
| --- | --- | --- | --- | --- | --- | --- | --- | --- | --- | --- |

Please continue to the next page…Please give your opinion about the **causes of Taylor Devani’s successes** by assigning a percentage to each of the following four causes, such that the four causes together **sum to 100%**.

% due to Taylor’s abilities and personality

| □  0 | □  5 | □  10 | □  15 | □  20 | □  25 | □  30 | □  35 | □  40 | □  45 | □  50 | □  55 | □  60 | □  65 | □  70 | □  75 | □  80 | □  85 | □  90 | □  95 | □  100 |
| --- | --- | --- | --- | --- | --- | --- | --- | --- | --- | --- | --- | --- | --- | --- | --- | --- | --- | --- | --- | --- |

% due to the amount of effort and attention Taylor applied

| □  0 | □  5 | □  10 | □  15 | □  20 | □  25 | □  30 | □  35 | □  40 | □  45 | □  50 | □  55 | □  60 | □  65 | □  70 | □  75 | □  80 | □  85 | □  90 | □  95 | □  100 |
| --- | --- | --- | --- | --- | --- | --- | --- | --- | --- | --- | --- | --- | --- | --- | --- | --- | --- | --- | --- | --- |

% due to Taylor’s job responsibilities, DeltaCom’s expectations, and the resources provided

| □  0 | □  5 | □  10 | □  15 | □  20 | □  25 | □  30 | □  35 | □  40 | □  45 | □  50 | □  55 | □  60 | □  65 | □  70 | □  75 | □  80 | □  85 | □  90 | □  95 | □  100 |
| --- | --- | --- | --- | --- | --- | --- | --- | --- | --- | --- | --- | --- | --- | --- | --- | --- | --- | --- | --- | --- |

% due to chance and random luck

| □  0 | □  5 | □  10 | □  15 | □  20 | □  25 | □  30 | □  35 | □  40 | □  45 | □  50 | □  55 | □  60 | □  65 | □  70 | □  75 | □  80 | □  85 | □  90 | □  95 | □  100 |
| --- | --- | --- | --- | --- | --- | --- | --- | --- | --- | --- | --- | --- | --- | --- | --- | --- | --- | --- | --- | --- |

**PLEASE CHECK: Do the above four numbers add to 100%? If not, please revise.**

Please give your opinion about the **causes of Taylor Devani’s failures** by assigning a percentage to each of the following four causes, such that the four causes together **sum to 100%**.

% due to Taylor’s abilities and personality

| □  0 | □  5 | □  10 | □  15 | □  20 | □  25 | □  30 | □  35 | □  40 | □  45 | □  50 | □  55 | □  60 | □  65 | □  70 | □  75 | □  80 | □  85 | □  90 | □  95 | □  100 |
| --- | --- | --- | --- | --- | --- | --- | --- | --- | --- | --- | --- | --- | --- | --- | --- | --- | --- | --- | --- | --- |

% due to the amount of effort and attention Taylor applied

| □  0 | □  5 | □  10 | □  15 | □  20 | □  25 | □  30 | □  35 | □  40 | □  45 | □  50 | □  55 | □  60 | □  65 | □  70 | □  75 | □  80 | □  85 | □  90 | □  95 | □  100 |
| --- | --- | --- | --- | --- | --- | --- | --- | --- | --- | --- | --- | --- | --- | --- | --- | --- | --- | --- | --- | --- |

% due to Taylor’s job responsibilities, DeltaCom’s expectations, and the resources provided

| □  0 | □  5 | □  10 | □  15 | □  20 | □  25 | □  30 | □  35 | □  40 | □  45 | □  50 | □  55 | □  60 | □  65 | □  70 | □  75 | □  80 | □  85 | □  90 | □  95 | □  100 |
| --- | --- | --- | --- | --- | --- | --- | --- | --- | --- | --- | --- | --- | --- | --- | --- | --- | --- | --- | --- | --- |

% due to chance and random luck

| □  0 | □  5 | □  10 | □  15 | □  20 | □  25 | □  30 | □  35 | □  40 | □  45 | □  50 | □  55 | □  60 | □  65 | □  70 | □  75 | □  80 | □  85 | □  90 | □  95 | □  100 |
| --- | --- | --- | --- | --- | --- | --- | --- | --- | --- | --- | --- | --- | --- | --- | --- | --- | --- | --- | --- | --- |

**PLEASE CHECK: Do the above four numbers add to 100%? If not, please revise.**

Please continue to the next page… Please indicate the extent to which you agree with the following statements about the **feedback session** (1= strongly disagree to 7 = strongly agree).

Strongly

disagree

Slightly

disagree

Slightly

agree

Strongly

agree

| The feedback discussion focused mostly on Taylor’s future behaviour. | 1 | 2 | 3 | 4 | 5 | 6 | 7 |
| --- | --- | --- | --- | --- | --- | --- | --- |
| You and Taylor came to agreement. | 1 | 2 | 3 | 4 | 5 | 6 | 7 |
| The feedback Taylor received was appropriate for the work completed. | 1 | 2 | 3 | 4 | 5 | 6 | 7 |
| You treated Taylor in a polite manner. | 1 | 2 | 3 | 4 | 5 | 6 | 7 |
| Based on the feedback, Taylor is now motivated to change behaviour. | 1 | 2 | 3 | 4 | 5 | 6 | 7 |
| At the end of the meeting, you and Taylor had similar views. | 1 | 2 | 3 | 4 | 5 | 6 | 7 |
| The feedback was justified, given Taylor’s performance. | 1 | 2 | 3 | 4 | 5 | 6 | 7 |
| You refrained from improper remarks or comments. | 1 | 2 | 3 | 4 | 5 | 6 | 7 |
| You and Taylor spent a large part of this session generating new ideas for Taylor’s next steps. | 1 | 2 | 3 | 4 | 5 | 6 | 7 |
| Taylor sees the value of acting on your suggestions. | 1 | 2 | 3 | 4 | 5 | 6 | 7 |
| You treated Taylor with respect. | 1 | 2 | 3 | 4 | 5 | 6 | 7 |
| The feedback you gave Taylor reflected what Taylor has contributed to DeltaCom. | 1 | 2 | 3 | 4 | 5 | 6 | 7 |
| You will probably recommend Taylor for promotion based on the feedback session. | 1 | 2 | 3 | 4 | 5 | 6 | 7 |
| You and Taylor now share the same opinion about what Taylor needs to do to be successful. | 1 | 2 | 3 | 4 | 5 | 6 | 7 |
| You treated Taylor with dignity. | 1 | 2 | 3 | 4 | 5 | 6 | 7 |
| Taylor will likely change behaviour, based on the feedback received. | 1 | 2 | 3 | 4 | 5 | 6 | 7 |
| Your feedback to Taylor accurately reflected the effort Taylor has put into the work. | 1 | 2 | 3 | 4 | 5 | 6 | 7 |
| The feedback conversation centred on what will make Taylor most successful going forward. | 1 | 2 | 3 | 4 | 5 | 6 | 7 |

Please continue to the next page…

**Thank you for playing Chris Sinopoli in this exercise.**

**You may now return to being yourself.**

Please print your name here: ­­__

The name of the person playing District Manager Taylor Devani: _______________________

**Before you go, a few last questions about you:**

Are you Male or Female? (Circle one)

What is your age? ___________________

What is the highest level of education you have completed? _______________________

What is the nature of your **current employment or most recent full-time job?** Circle the one that fits best:

○ Front-line employee ○ Self-employed individual ○ Full-time student

○ Professional practice ○ Entrepreneur/small business

○ Junior management ○ Middle management ○ Executive/upper management

What country did you live in the most between ages 12 and 18?

_________________________________________

**Please place your instruction pages and this questionnaire into the envelope provided, together with those of your role-play partner. Then, when you are both finished, hand the envelope to the assistant.**

**Thank you!**
