# Supplementary material for: The future of feedback: Motivating performance improvement through future-focused feedback
Source: PLoS One. 2020 Jun 19;15(6):e0234444. doi: 10.1371/journal.pone.0234444 (PMC7304587; doi:10.1371/journal.pone.0234444)
Supplement: S10 Text — (DOCX) [file pone.0234444.s010.docx]

**The future of feedback: Motivating performance improvement**

**through future-focused feedback**

Jackie Gnepp, Joshua Klayman, Ian O. Williamson, Sema Barlas

**S14 Text. Study 3 post-discussion questionnaire – District Manager.**

**DELTACOM CORPORATION EXERCISE**

**Please answer *every* question asked below. Please continue in your role as Taylor Devani, and provide Taylor’s answers to these questions.**

Please rate the **content** of the feedback that Chris Sinopoli gave you

from **0 = almost all negative** to **10 = almost all positive**.

Almost all negative Equal Almost all positive

| □  0 | □  1 | □  2 | □  3 | □  4 | □  5 | □  6 | □  7 | □  8 | □  9 | □  10 |
| --- | --- | --- | --- | --- | --- | --- | --- | --- | --- | --- |

Please rate the accuracy of the feedback that Chris Sinopoli gave you

from **0%** to **100% accurate**.

| □  0 | □  5 | □  10 | □  15 | □  20 | □  25 | □  30 | □  35 | □  40 | □  45 | □  50 | □  55 | □  60 | □  65 | □  70 | □  75 | □  80 | □  85 | □  90 | □  95 | □  100 |
| --- | --- | --- | --- | --- | --- | --- | --- | --- | --- | --- | --- | --- | --- | --- | --- | --- | --- | --- | --- | --- |

Please rate how qualified Chris Sinopoli was to give you feedback

from **0 = unqualified** to **10 = completely qualified**.

| □  0 | □  1 | □  2 | □  3 | □  4 | □  5 | □  6 | □  7 | □  8 | □  9 | □  10 |
| --- | --- | --- | --- | --- | --- | --- | --- | --- | --- | --- |

Please continue to the next page…Please give your opinion about the **causes of your successes** by assigning a percentage to each of the following four causes, such that the four causes together **sum to 100%**.

% due to your abilities and personality

| □  0 | □  5 | □  10 | □  15 | □  20 | □  25 | □  30 | □  35 | □  40 | □  45 | □  50 | □  55 | □  60 | □  65 | □  70 | □  75 | □  80 | □  85 | □  90 | □  95 | □  100 |
| --- | --- | --- | --- | --- | --- | --- | --- | --- | --- | --- | --- | --- | --- | --- | --- | --- | --- | --- | --- | --- |

% due to the amount of effort and attention you applied

| □  0 | □  5 | □  10 | □  15 | □  20 | □  25 | □  30 | □  35 | □  40 | □  45 | □  50 | □  55 | □  60 | □  65 | □  70 | □  75 | □  80 | □  85 | □  90 | □  95 | □  100 |
| --- | --- | --- | --- | --- | --- | --- | --- | --- | --- | --- | --- | --- | --- | --- | --- | --- | --- | --- | --- | --- |

% due to your job responsibilities, DeltaCom’s expectations, and the resources provided

| □  0 | □  5 | □  10 | □  15 | □  20 | □  25 | □  30 | □  35 | □  40 | □  45 | □  50 | □  55 | □  60 | □  65 | □  70 | □  75 | □  80 | □  85 | □  90 | □  95 | □  100 |
| --- | --- | --- | --- | --- | --- | --- | --- | --- | --- | --- | --- | --- | --- | --- | --- | --- | --- | --- | --- | --- |

% due to chance and random luck

| □  0 | □  5 | □  10 | □  15 | □  20 | □  25 | □  30 | □  35 | □  40 | □  45 | □  50 | □  55 | □  60 | □  65 | □  70 | □  75 | □  80 | □  85 | □  90 | □  95 | □  100 |
| --- | --- | --- | --- | --- | --- | --- | --- | --- | --- | --- | --- | --- | --- | --- | --- | --- | --- | --- | --- | --- |

**PLEASE CHECK: Do the above four numbers add to 100%? If not, please revise.**

Please give your opinion about the **causes of your failures** by assigning a percentage to each of the following four causes, such that the four causes together **sum to 100%**.

% due to your abilities and personality

| □  0 | □  5 | □  10 | □  15 | □  20 | □  25 | □  30 | □  35 | □  40 | □  45 | □  50 | □  55 | □  60 | □  65 | □  70 | □  75 | □  80 | □  85 | □  90 | □  95 | □  100 |
| --- | --- | --- | --- | --- | --- | --- | --- | --- | --- | --- | --- | --- | --- | --- | --- | --- | --- | --- | --- | --- |

% due to the amount of effort and attention you applied

| □  0 | □  5 | □  10 | □  15 | □  20 | □  25 | □  30 | □  35 | □  40 | □  45 | □  50 | □  55 | □  60 | □  65 | □  70 | □  75 | □  80 | □  85 | □  90 | □  95 | □  100 |
| --- | --- | --- | --- | --- | --- | --- | --- | --- | --- | --- | --- | --- | --- | --- | --- | --- | --- | --- | --- | --- |

% due to your job responsibilities, DeltaCom’s expectations, and the resources provided

| □  0 | □  5 | □  10 | □  15 | □  20 | □  25 | □  30 | □  35 | □  40 | □  45 | □  50 | □  55 | □  60 | □  65 | □  70 | □  75 | □  80 | □  85 | □  90 | □  95 | □  100 |
| --- | --- | --- | --- | --- | --- | --- | --- | --- | --- | --- | --- | --- | --- | --- | --- | --- | --- | --- | --- | --- |

% due to chance and random luck

| □  0 | □  5 | □  10 | □  15 | □  20 | □  25 | □  30 | □  35 | □  40 | □  45 | □  50 | □  55 | □  60 | □  65 | □  70 | □  75 | □  80 | □  85 | □  90 | □  95 | □  100 |
| --- | --- | --- | --- | --- | --- | --- | --- | --- | --- | --- | --- | --- | --- | --- | --- | --- | --- | --- | --- | --- |

**PLEASE CHECK: Do the above four numbers add to 100%? If not, please revise.**

## Please continue to the next page… Please indicate the extent to which you agree with the following statements about the feedback session (1= strongly disagree to 7 = strongly agree).

Strongly

disagree

Slightly

disagree

Slightly

agree

Strongly

agree

| The feedback discussion focused mostly on your future behaviour. | 1 | 2 | 3 | 4 | 5 | 6 | 7 |
| --- | --- | --- | --- | --- | --- | --- | --- |
| You and Chris came to agreement. | 1 | 2 | 3 | 4 | 5 | 6 | 7 |
| The feedback you received was appropriate for the work you have completed. | 1 | 2 | 3 | 4 | 5 | 6 | 7 |
| Chris treated you in a polite manner. | 1 | 2 | 3 | 4 | 5 | 6 | 7 |
| Based on the feedback, you are now motivated to change your behaviour. | 1 | 2 | 3 | 4 | 5 | 6 | 7 |
| At the end of the meeting, you and Chris had similar views. | 1 | 2 | 3 | 4 | 5 | 6 | 7 |
| The feedback was justified, given your performance. | 1 | 2 | 3 | 4 | 5 | 6 | 7 |
| Chris refrained from improper remarks or comments. | 1 | 2 | 3 | 4 | 5 | 6 | 7 |
| You and Chris spent a large part of this session generating new ideas for your next steps. | 1 | 2 | 3 | 4 | 5 | 6 | 7 |
| You see the value of acting on Chris’s suggestions. | 1 | 2 | 3 | 4 | 5 | 6 | 7 |
| Chris treated you with respect. | 1 | 2 | 3 | 4 | 5 | 6 | 7 |
| The feedback Chris gave you reflected what you have contributed to DeltaCom. | 1 | 2 | 3 | 4 | 5 | 6 | 7 |
| Chris will probably recommend you for promotion based on the feedback session. | 1 | 2 | 3 | 4 | 5 | 6 | 7 |
| You and Chris now share the same opinion about what you need to do to be successful. | 1 | 2 | 3 | 4 | 5 | 6 | 7 |
| Chris treated you with dignity. | 1 | 2 | 3 | 4 | 5 | 6 | 7 |
| You will likely change your behaviour, based on the feedback received. | 1 | 2 | 3 | 4 | 5 | 6 | 7 |
| Chris’s feedback to you accurately reflected the effort you have put into your work. | 1 | 2 | 3 | 4 | 5 | 6 | 7 |
| The feedback conversation centred on what will make you most successful going forward. | 1 | 2 | 3 | 4 | 5 | 6 | 7 |

Please continue to the next page…

**Thank you for playing Taylor Devani in this exercise.**

**You may now return to being yourself.**

Please print your name here: ­­__

The name of the person playing Regional Manager Chris Sinopoli: _______________________

**Before you go, a few last questions about you:**

Are you Male or Female? (Circle one)

What is your age? ___________________

What is the highest level of education you have completed? _______________________

What is the nature of your **current employment or most recent full-time job?** Circle the one that fits best:

○ Front-line employee ○ Self-employed individual ○ Full-time student

○ Professional practice ○ Entrepreneur/small business

○ Junior management ○ Middle management ○ Executive/upper management

What country did you live in the most between ages 12 and 18?

_________________________________________

**Please place your instruction pages and this questionnaire into the envelope provided, together with those of your role-play partner. Then, when you are both finished, hand the envelope to the assistant.**

**Thank you!**
