# Supplementary material for: The future of feedback: Motivating performance improvement through future-focused feedback
Source: PLoS One. 2020 Jun 19;15(6):e0234444. doi: 10.1371/journal.pone.0234444 (PMC7304587; doi:10.1371/journal.pone.0234444)
Supplement: S11 Text — (DOCX) [file pone.0234444.s011.docx]

**The future of feedback: Motivating performance improvement**

**through future-focused feedback**

Jackie Gnepp, Joshua Klayman, Ian O. Williamson, Sema Barlas

**S16 Text. Hypothetical example of future-focused feedback.**

The article opens with this vignette:

Once again, Taylor Devani is hoping to be promoted to Regional Manager. Chris Sinopoli, Taylor’s new boss, has arranged a meeting to provide performance feedback, especially regarding ways Taylor must change to succeed in a Regional Manager position. Like Taylor’s previous boss, Chris is delighted with Taylor’s award-winning sales performance. But Taylor was admonished in last year’s performance appraisal about cavalier treatment of customers and intolerant behavior toward employees. Taylor was very resistant to that message then and there have been no noticeable improvements since. What can Chris say to get through to Taylor?

The following is a possible future-focused response:

- Express the goal of improving future performance (e.g., “Taylor, I’d like to provide feedback that will be useful in preparing you for an eventual promotion to Regional Manager.”)
- Specify performance standards and ideals (e.g., “DeltaCom is looking to promote managers who excel in all areas of their job descriptions and show the potential to lead larger teams.”)
- Review past performance, providing warranted praise and, where performance is poor, sticking to the facts and avoiding discussion of causes and explanations (e.g., “Your sales have been incredible; we’re all very proud of you… Turnover of your direct reports is higher than we would like to see.”)
- Assume motivation and competence to improve (e.g., “I realize there are only 24 hours in a day and you have been focused on generating high sales for the company, but I believe you have what it takes to improve customer service and develop your people too.”)
- Invite discussion of plans and milestones (e.g., “What ideas do you have for how to strengthen customer relations? Is there any way to combine that with your goal of developing your employees? How might DeltaCom facilitate that? What’s a reasonable target for 6 months from now?”)
- Develop solutions together (e.g., “I like your idea to … but I can imagine your running into difficulties if you … Would it be helpful if I …?)
