# Supplementary material for: The future of feedback: Motivating performance improvement through future-focused feedback
Source: PLoS One. 2020 Jun 19;15(6):e0234444. doi: 10.1371/journal.pone.0234444 (PMC7304587; doi:10.1371/journal.pone.0234444)
Supplement: S2 Table — (DOCX) [file pone.0234444.s013.docx]

**The future of feedback: Motivating performance improvement**

Jackie Gnepp, Joshua Klayman, Ian O. Williamson, Sema Barlas

**S10 Table. Study 2 means and standard deviations**

**for evaluations of successes and failures, by role.**

|  | Providers | | Recipients | |
| --- | --- | --- | --- | --- |
|  | Mean | s.d. | Mean | s.d. |
| Quality-success | 6.6 | 0.6 | 6.9 | 0.4 |
| Importance-success | 6.2 | 0.9 | 6.4 | 0.9 |
| Quality-failure | 3.2 | 0.9 | 3.5 | 1.0 |
| Importance-failure | 5.5 | 1.4 | 5.6 | 1.1 |
| Internal-attirbution-success | 71.6 | 13.7 | 75.9 | 13.5 |
| Internal-attirbution-failure | 72.1 | 18.9 | 63.9 | 24.6 |
