# Supplementary material for: The future of feedback: Motivating performance improvement through future-focused feedback
Source: PLoS One. 2020 Jun 19;15(6):e0234444. doi: 10.1371/journal.pone.0234444 (PMC7304587; doi:10.1371/journal.pone.0234444)
Supplement: S1 Analyses — (PDF) [file pone.0234444.s015.pdf]

The future of feedback: Motivating performance improvement

Jackie Gnepp, Joshua Klayman, Ian O. Williamson, Sema Barlas

S9 Analyses. Study 2 ANOVAs with between-groups comparisons.

General Linear Model (SPSS)

Group = Pre-post or post-only **Role** = RM (Regional Manager--feedback provider) DM = (District Manger--feedback recipient). **succ_fail** = ratings for recipient's successes or for failures Note: T2 indicates after feedback discussion; pre-discussion data are not used here.

Percentage Internal Attributions (arcsine transformed)

```
GLM arcIntrnlSucRMT2 arcIntrnlFailRMT2 arcIntrnlSucDMT2 arcIntrnlFailDMT2 BY G
roup
  /WSFACTOR=role 2 Polynomial succ_fail 2 Polynomial
  /MEASURE=pcInternal
  /METHOD=SSTYPE(3)
  /EMMEANS=TABLES(OVERALL)
  /EMMEANS=TABLES(Group)
  /EMMEANS=TABLES(role)
  /EMMEANS=TABLES(succ_fail)
  /EMMEANS=TABLES(Group*role)
  /EMMEANS=TABLES(Group*succ_fail)
  /EMMEANS=TABLES(role*succ_fail)
  /EMMEANS=TABLES(Group*role*succ_fail)
  /PRINT=ETASQ
  /CRITERIA=ALPHA(.05)
  /WSDESIGN=role succ_fail role*succ_fail
  /DESIGN=Group.
```

Within-Subjects Factors

Measure: pcInternal

role	succ_fail	Dependent Variable
1	1	arcIntrnlSucRMT2
	2	arcIntrnlFailRMT2
2	1	arcIntrnlSucDMT2
	2	arcIntrnlFailDMT2

Between-Subjects Factors

		Value Label	N
Group	1	use pre-post	117
	2	use post-only	68

Tests of Within-Subjects Contrasts

Measure: pcInternal

Source	role	succ_fail	Type III Sum of Squares	df	Mean Square	F
role	Linear		.215	1	.215	1.120
role * Group	Linear		.021	1	.021	.108
Error(role)	Linear		35.071	183	.192	
succ_fail		Linear	3.008	1	3.008	19.256
succ_fail * Group		Linear	.033	1	.033	.213
Error(succ_fail)		Linear	28.585	183	.156	
role * succ_fail	Linear	Linear	1.947	1	1.947	12.208
role * succ_fail * Group	Linear	Linear	.377	1	.377	2.365
Error(role*succ_fail)	Linear	Linear	29.189	183	.160	

Tests of Within-Subjects Contrasts

Measure: pcInternal

Source	role	succ_fail	Sig.	Partial Eta Squared
role	Linear		.291	.006
role * Group	Linear		.742	.001
Error(role)	Linear			
succ_fail		Linear	.000	.095
succ_fail * Group		Linear	.645	.001
Error(succ_fail)		Linear		
role * succ_fail	Linear	Linear	.001	.063
role * succ_fail * Group	Linear	Linear	.126	.013
Error(role*succ_fail)	Linear	Linear		

Tests of Between-Subjects Effects

Measure: pcInternal

Transformed Variable: Average

Source	Type III Sum of Squares	df	Mean Square	F	Sig.	Partial Eta Squared
Intercept	2870.102	1	2870.102	13066.819	.000	.986
Group	.003	1	.003	.013	.911	.000
Error	40.196	183	.220			

Estimated Marginal Means

1. Grand Mean

Measure: pcInternal

Mean	Std. Error	95% Confidence Interval	
		Lower Bound	Upper Bound
2.042	.018	2.007	2.078

2. 1 =q'aries pre & post; 2 = q'aire post only

Measure: pcInternal

1 =q'aries pre & post; 2 = q'aire post only	Mean	Std. Error	95% Confidence Interval	
			Lower Bound	Upper Bound
1	2.040	.022	1.998	2.083
2	2.044	.028	1.988	2.100

3. role

Measure: pcInternal

role	Mean	Std. Error	95% Confidence Interval	
			Lower Bound	Upper Bound
1	2.060	.024	2.012	2.108
2	2.025	.025	1.976	2.073

4. succ_fail

Measure: pcInternal

succ_fail	Mean	Std. Error	95% Confidence Interval	
			Lower Bound	Upper Bound
1	2.108	.016	2.076	2.141
2	1.976	.029	1.920	2.033

5. 1 =q'aries pre & post; 2 = q'aire post only * role

Measure: pcInternal

1 =q'aries pre & post; 2 = q'aire post only	role	Mean	Std. Error	95% Confidence Interval	
				Lower Bound	Upper Bound
1	1	2.063	.029	2.006	2.121
	2	2.017	.030	1.958	2.076
2	1	2.057	.039	1.981	2.132
	2	2.032	.039	1.955	2.110

6. 1 =q'aries pre & post; 2 = q'aire post only * succ_fail

Measure: pcInternal

1 =q'aries pre & post; 2 = q'aire post only	succ_fail	Mean	Std. Error	95% Confidence Interval	
				Lower Bound	Upper Bound
1	1	2.099	.020	2.060	2.139
	2	1.981	.035	1.913	2.050
2	1	2.117	.026	2.066	2.169
	2	1.971	.046	1.881	2.061

7. role * succ_fail

Measure: pcInternal

role	succ_fail	Mean	Std. Error	95% Confidence Interval	
				Lower Bound	Upper Bound
1	1	2.073	.025	2.025	2.121
	2	2.047	.035	1.977	2.117
2	1	2.144	.025	2.094	2.194
	2	1.905	.042	1.823	1.987

8. 1 =q'aries pre & post; 2 = q'aire post only * role * succ_fail

Measure: pcInternal

1 =q'aries pre & post; 2 = q'aire post only	role	succ_fail	Mean	Std. Error	95% Confidence Interval	
					Lower Bound	Upper Bound
1	1	1	2.046	.030	1.987	2.105
		2	2.081	.043	1.996	2.166
	2	1	2.153	.031	2.092	2.213
		2	1.881	.050	1.782	1.981
2	1	1	2.100	.039	2.023	2.177
		2	2.013	.056	1.902	2.124
	2	1	2.135	.040	2.056	2.214
		2	1.929	.066	1.799	2.060

Performance quality ratings

SalesPerf = rating for sales, i.e. successes; **LowPerfs** = average rating for low-performance aspects, i.e. failures

```
GLM SalesPerfRMT2 LowPerfsRMT2 SalesPerfDMT2 LowPerfsDMT2 BY Wave
  /WSFACTOR=role 2 Polynomial succ_fail 2 Polynomial
  /MEASURE=PerformanceQuality
  /METHOD=SSTYPE(3)
  /EMMEANS=TABLES(OVERALL)
```

```
/EMMEANS=TABLES(Group)
/EMMEANS=TABLES(role)
/EMMEANS=TABLES(succ_fail)
/EMMEANS=TABLES(Group*role)
/EMMEANS=TABLES(Group*succ_fail)
/EMMEANS=TABLES(role*succ_fail)
/EMMEANS=TABLES(Group*role*succ_fail)
/PRINT=ETASQ
/CRITERIA=ALPHA(.05)
/WSDESIGN=role succ_fail role*succ_fail
/DESIGN=Group.
```

Within-Subjects Factors

Measure: PerformanceQuality

role	succ_fail	Dependent Variable
1	1	SalesPerfRMT2
	2	LowPerfsRMT2
2	1	SalesPerfDMT2
	2	LowPerfsDMT2

Between-Subjects Factors

		Value Label	N
Group	1	use pre-post	116
	2	use post-only	66

Tests of Within-Subjects Contrasts

Measure: PerformanceQuality

Source	role	succ_fail	Type III Sum of Squares	df	Mean Square	F
role	Linear		5.935	1	5.935	10.677
role * Group	Linear		1.305	1	1.305	2.347
Error(role)	Linear		100.050	180	.556	
succ_fail		Linear	1964.996	1	1964.996	3231.344
succ_fail * Group		Linear	.019	1	.019	.032
Error(succ_fail)		Linear	109.459	180	.608	
role * succ_fail	Linear	Linear	.241	1	.241	.449
role * succ_fail * Group	Linear	Linear	.032	1	.032	.060
Error(role*succ_fail)	Linear	Linear	96.686	180	.537	

Tests of Within-Subjects Contrasts

Measure: PerformanceQuality

Source	role	succ_fail	Sig.	Partial Eta Squared
role	Linear		.001	.056
role * Group	Linear		.127	.013
Error(role)	Linear			
succ_fail		Linear	.000	.947
succ_fail * Group		Linear	.859	.000
Error(succ_fail)		Linear		
role * succ_fail	Linear	Linear	.504	.002
role * succ_fail * Group	Linear	Linear	.806	.000
Error(role*succ_fail)	Linear	Linear		

Tests of Between-Subjects Effects

Measure: PerformanceQuality

Transformed Variable: Average

Source	Type III Sum of Squares	df	Mean Square	F	Sig.	Partial Eta Squared
Intercept	16849.678	1	16849.678	25072.582	.000	.993
Group	1.258	1	1.258	1.872	.173	.010
Error	120.966	180	.672			

Estimated Marginal Means

1. Grand Mean

Measure: PerformanceQuality

Mean	Std. Error	95% Confidence Interval	
		Lower Bound	Upper Bound
5.003	.032	4.941	5.066

2. 1 =q'aries pre & post; 2 = q'aire post only

Measure: PerformanceQuality

1 =q'aries pre & post; 2 = q'aire post only	Mean	Std. Error	95% Confidence Interval	
			Lower Bound	Upper Bound
1	5.047	.038	4.972	5.122
2	4.960	.050	4.861	5.060

3. role

Measure: PerformanceQuality

role	Mean	Std. Error	95% Confidence Interval	
			Lower Bound	Upper Bound
1	4.910	.044	4.822	4.997
2	5.097	.041	5.016	5.179

4. succ_fail

Measure: PerformanceQuality

succ_fail	Mean	Std. Error	95% Confidence Interval	
			Lower Bound	Upper Bound
1	6.712	.034	6.646	6.778
2	3.295	.052	3.193	3.397

5. 1 =q'aries pre & post; 2 = q'aire post only * role

Measure: PerformanceQuality

1 =q'aries pre & post; 2 = q'aire post only	role	Mean	Std. Error	95% Confidence Interval	
				Lower Bound	Upper Bound
1	1	4.909	.053	4.804	5.014
	2	5.185	.050	5.087	5.283
2	1	4.910	.071	4.771	5.050
	2	5.010	.066	4.880	5.140

6. 1 =q'aries pre & post; 2 = q'aire post only * succ_fail

Measure: PerformanceQuality

1 =q'aries pre & post; 2 = q'aire post only	succ_fail	Mean	Std. Error	95% Confidence Interval	
				Lower Bound	Upper Bound
1	1	6.750	.040	6.670	6.830
	2	3.343	.062	3.220	3.466
2	1	6.674	.053	6.569	6.780
	2	3.246	.083	3.083	3.409

7. role * succ_fail

Measure: PerformanceQuality

role	succ_fail	Mean	Std. Error	95% Confidence Interval	
				Lower Bound	Upper Bound
1	1	6.637	.051	6.537	6.737
	2	3.182	.065	3.053	3.311
2	1	6.787	.041	6.706	6.868
	2	3.408	.075	3.261	3.555

8. 1 =q'aries pre & post; 2 = q'aire post only * role * succ_fail

Measure: PerformanceQuality

1 =q'aries pre & post; 2 = q'aire post only	role	succ_fail	Mean	Std. Error	95% Confidence Interval Lower Bound	95% Confidence Interval Upper Bound
1	1	1	6.638	.061	6.517	6.759
		2	3.180	.079	3.024	3.335
	2	1	6.862	.050	6.764	6.960
		2	3.507	.090	3.330	3.684
2	1	1	6.636	.081	6.476	6.796
		2	3.184	.104	2.978	3.390
	2	1	6.712	.066	6.582	6.842
		2	3.308	.119	3.073	3.543

Performance importance ratings

. **SalesImp** = rating for sales, i.e. successes; **LowPerfsImp** = average rating for low-performance aspects, i.e. failures

```
GLM SaleImpRMT2 LowPerfsImpRMT2 SaleImpDMT2 LowPerfsImpDMT2 BY Wave
  /WSFACTOR=role 2 Polynomial succ_fail 2 Polynomial
  /MEASURE=PerformanceImportance
  /METHOD=SSTYPE(3)
  /EMMEANS=TABLES(OVERALL)
  /EMMEANS=TABLES(Group)
  /EMMEANS=TABLES(role)
  /EMMEANS=TABLES(succ_fail)
  /EMMEANS=TABLES(Group*role)
  /EMMEANS=TABLES(Group*succ_fail)
  /EMMEANS=TABLES(role*succ_fail)
  /EMMEANS=TABLES(Group*role*succ_fail)
  /PRINT=ETASQ
  /CRITERIA=ALPHA(.05)
  /WSDESIGN=role succ_fail role*succ_fail
  /DESIGN=Group.
```

Within-Subjects Factors

Measure: PerformanceImportance

role	succ_fail	Dependent Variable
1	1	SaleImpRMT2
	2	LowPerfsImp RMT2
2	1	SaleImpDMT2
	2	LowPerfsImp DMT2

Between-Subjects Factors

		Value Label	N
Group	1	use pre-post	115
	2	use post-only	67

Tests of Within-Subjects Contrasts

Measure: PerformanceImportance

Source	role	succ_fail	Type III Sum of Squares	df	Mean Square	F
role	Linear		1.939	1	1.939	2.047
role * Group	Linear		.532	1	.532	.561
Error(role)	Linear		170.505	180	.947	
succ_fail		Linear	97.615	1	97.615	64.782
succ_fail * Group		Linear	.133	1	.133	.089
Error(succ_fail)		Linear	271.227	180	1.507	
role * succ_fail	Linear	Linear	1.547	1	1.547	1.377
role * succ_fail * Group	Linear	Linear	.179	1	.179	.160
Error(role*succ_fail)	Linear	Linear	202.179	180	1.123	

Tests of Within-Subjects Contrasts

Measure: PerformanceImportance

Source	role	succ_fail	Sig.	Partial Eta Squared
role	Linear		.154	.011
role * Group	Linear		.455	.003
Error(role)	Linear			
succ_fail		Linear	.000	.265
succ_fail * Group		Linear	.766	.000
Error(succ_fail)		Linear		
role * succ_fail	Linear	Linear	.242	.008
role * succ_fail * Group	Linear	Linear	.690	.001
Error(role*succ_fail)	Linear	Linear		

Tests of Between-Subjects Effects

Measure: PerformanceImportance

Transformed Variable: Average

Source	Type III Sum of Squares	df	Mean Square	F	Sig.	Partial Eta Squared
Intercept	24320.790	1	24320.790	27613.623	.000	.994
Group	2.605	1	2.605	2.958	.087	.016
Error	158.536	180	.881			

Estimated Marginal Means

1. Grand Mean

Measure: PerformanceImportance

Mean	Std. Error	95% Confidence Interval	
		Lower Bound	Upper Bound
5.992	.036	5.921	6.063

2. 1 =q'aries pre & post; 2 = q'aire post only

Measure: PerformanceImportance

1 =q'aries pre & post; 2 = q'aire post only	Mean	Std. Error	95% Confidence Interval	
			Lower Bound	Upper Bound
1	5.930	.044	5.844	6.016
2	6.054	.057	5.941	6.167

3. role

Measure: PerformanceImportance

role	Mean	Std. Error	95% Confidence Interval	
			Lower Bound	Upper Bound
1	5.939	.056	5.828	6.049
2	6.046	.048	5.952	6.140

4. succ_fail

Measure: PerformanceImportance

succ_fail	Mean	Std. Error	95% Confidence Interval	
			Lower Bound	Upper Bound
1	6.372	.047	6.278	6.465
2	5.612	.069	5.476	5.749

5. 1 =q'aries pre & post; 2 = q'aire post only * role

Measure: PerformanceImportance

1 =q'aries pre & post; 2 = q'aire post only	role	Mean	Std. Error	95% Confidence Interval	
				Lower Bound	Upper Bound
1	1	5.849	.068	5.715	5.982
	2	6.012	.058	5.897	6.126
2	1	6.029	.089	5.853	6.204
	2	6.080	.076	5.930	6.229

6. 1 =q'aries pre & post; 2 = q'aire post only * succ_fail

Measure: PerformanceImportance

1 =q'aries pre & post; 2 = q'aire post only	succ_fail	Mean	Std. Error	95% Confidence Interval	
				Lower Bound	Upper Bound
1	1	6.296	.058	6.182	6.409
	2	5.564	.084	5.399	5.730
2	1	6.448	.075	6.299	6.596
	2	5.660	.110	5.443	5.878

7. role * succ_fail

Measure: PerformanceImportance

role	succ_fail	Mean	Std. Error	95% Confidence Interval	
				Lower Bound	Upper Bound
1	1	6.270	.067	6.138	6.402
	2	5.607	.101	5.407	5.806
2	1	6.473	.067	6.341	6.605
	2	5.618	.085	5.451	5.785

8. 1 =q'aries pre & post; 2 = q'aire post only * role * succ_fail

Measure: PerformanceImportance

1 =q'aries pre & post; 2 = q'aire post only	role	succ_fail	Mean	Std. Error	95% Confidence Interval	
					Lower Bound	Upper Bound
1	1	1	6.183	.081	6.022	6.343
		2	5.514	.123	5.272	5.757
	2	1	6.409	.081	6.248	6.569
		2	5.614	.103	5.412	5.817
2	1	1	6.358	.106	6.148	6.568
		2	5.699	.161	5.382	6.016
	2	1	6.537	.106	6.327	6.747
		2	5.622	.134	5.357	5.887

Ratings of feedback accuracy (FeedAcc), qualifications of provider (Qualified), and favorability of feedback (FeedContent)

```
GLM FeedAccRMT2 FeedAccDMT2 QualifiedRMT2 QualifiedDMT2 FeedContentRMT2 FeedCo
ntentDMT2 BY Group
  /WSFACTOR=RoleRM_DM 2 Polynomial
  /MEASURE=Accuracy Qualification Favorability
  /METHOD=SSTYPE(3)
  /EMMEANS=TABLES(OVERALL)
  /EMMEANS=TABLES(Group)
  /EMMEANS=TABLES(RoleRM_DM)
  /EMMEANS=TABLES(Group*RoleRM_DM)
  /PRINT=DESCRIPTIVE ETASQ
  /CRITERIA=ALPHA(.05)
  /WSDESIGN=RoleRM_DM
  /DESIGN=Group.
```

Within-Subjects Factors

Measure	RoleRM_DM	Dependent Variable
Accuracy	1	FeedAccRMT2
	2	FeedAccDMT2
Qualification	1	QualifiedRMT2
	2	QualifiedDMT2
Favorability	1	FeedContentRMT2
	2	FeedContentDMT2

Between-Subjects Factors

		Value Label	N
Group	1	use pre-post	117
	2	use post-only	67

Tests of Within-Subjects Contrasts

Source	Measure	RoleRM_DM	Type III Sum of Squares	df	Mean Square
RoleRM_DM	Accuracy	Linear	3.786	1	3.786
	Qualification	Linear	12.010	1	12.010
	Favorability	Linear	.298	1	.298
RoleRM_DM * Group	Accuracy	Linear	522.265	1	522.265
	Qualification	Linear	4.184	1	4.184
	Favorability	Linear	.811	1	.811
Error(RoleRM_DM)	Accuracy	Linear	50720.602	182	278.685
	Qualification	Linear	835.675	182	4.592
	Favorability	Linear	431.341	182	2.370

Tests of Within-Subjects Contrasts

Source	Measure	RoleRM_DM	F	Sig.	Partial Eta Squared
RoleRM_DM	Accuracy	Linear	.014	.907	.000
	Qualification	Linear	2.616	.108	.014
	Favorability	Linear	.126	.723	.001
RoleRM_DM * Group	Accuracy	Linear	1.874	.173	.010
	Qualification	Linear	.911	.341	.005
	Favorability	Linear	.342	.559	.002
Error(RoleRM_DM)	Accuracy	Linear			
	Qualification	Linear			
	Favorability	Linear			

Tests of Between-Subjects Effects

Transformed Variable: Average

Source	Measure	Type III Sum of Squares	df	Mean Square	F	Sig.
Intercept	Accuracy	1972328.971	1	1972328.971	5704.356	.000
	Qualification	8810.909	1	8810.909	1556.807	.000
	Favorability	9778.965	1	9778.965	2226.978	.000
Group	Accuracy	214.840	1	214.840	.621	.432
	Qualification	5.692	1	5.692	1.006	.317
	Favorability	16.778	1	16.778	3.821	.052
Error	Accuracy	62928.027	182	345.758		
	Qualification	1030.048	182	5.660		
	Favorability	799.187	182	4.391		

Tests of Between-Subjects Effects

Transformed Variable: Average

Source	Measure	Partial Eta Squared
Intercept	Accuracy	.969
	Qualification	.895
	Favorability	.924
Group	Accuracy	.003
	Qualification	.005
	Favorability	.021
Error	Accuracy	
	Qualification	
	Favorability	

Estimated Marginal Means

1. Grand Mean

Measure	Mean	Std. Error	95% Confidence Interval	
			Lower Bound	Upper Bound
Accuracy	76.072	1.007	74.084	78.059
Qualification	5.084	.129	4.830	5.339
Favorability	5.356	.114	5.133	5.580

2. 1 =q'aries pre & post; 2 = q'aire post only

Measure	1 =q'aries pre & post; 2 = q'aire post only	Mean	Std. Error	95% Confidence Interval	
				Lower Bound	Upper Bound
Accuracy	1	75.278	1.216	72.879	77.676
	2	76.866	1.606	73.696	80.035
Qualification	1	5.214	.156	4.907	5.521
	2	4.955	.206	4.550	5.361
Favorability	1	5.135	.137	4.864	5.405
	2	5.578	.181	5.221	5.936

3. RoleRM_DM

Measure	RoleRM_DM	Mean	Std. Error	95% Confidence Interval	
				Lower Bound	Upper Bound
Accuracy	1	75.966	1.136	73.724	78.208
	2	76.177	1.540	73.138	79.217
Qualification	1	5.272	.163	4.950	5.594
	2	4.897	.183	4.535	5.258
Favorability	1	5.386	.121	5.147	5.625
	2	5.327	.158	5.015	5.639

4. 1 =q'aries pre & post; 2 = q'aire post only * RoleRM_DM

Measure	1 =q'aries pre & post; 2 = q'aire post only	RoleRM_DM	Mean	Std. Error	95% ...
					Lower Bound
Accuracy	1	1	76.410	1.371	73.704
		2	74.145	1.859	70.477
	2	1	75.522	1.812	71.947
		2	78.209	2.457	73.362
Qualification	1	1	5.291	.197	4.902
		2	5.137	.221	4.701
	2	1	5.254	.260	4.741
		2	4.657	.292	4.080
Favorability	1	1	5.115	.146	4.827
		2	5.154	.191	4.777
	2	1	5.657	.193	5.276
		2	5.500	.252	5.002

4. 1 =q'aries pre & post; 2 = q'aire post only * RoleRM_DM

Measure	1 =q'aries pre & post; 2 = q'aire post only	RoleRM_DM	95% Confidence
			Upper Bound
Accuracy	1	1	79.116
		2	77.814
	2	1	79.098
		2	83.056
Qualification	1	1	5.679
		2	5.573
	2	1	5.767
		2	5.233
Favorability	1	1	5.403
		2	5.531
	2	1	6.037
		2	5.998
