# Supplementary material for: The future of feedback: Motivating performance improvement through future-focused feedback
Source: PLoS One. 2020 Jun 19;15(6):e0234444. doi: 10.1371/journal.pone.0234444 (PMC7304587; doi:10.1371/journal.pone.0234444)
Supplement: S2 Analyses — (DOCX) [file pone.0234444.s016.docx]

**The future of feedback: Motivating performance improvement**

**through future-focused feedback**

Jackie Gnepp, Joshua Klayman, Ian O. Williamson, Sema Barlas

**S15 Analyses. Studies 2 and 3 HLM analyses.**

| The SAS System |
| --- |

The Mixed Procedure

| **Model Information** | |
| --- | --- |
|  |  |
| **Dependent Variable** | Feedback Acceptance |
| **Covariance Structure** | Unstructured |
| **Subject Effect** | Study 2 vs 3 |
| **Estimation Method** | REML |
| **Residual Variance Method** | Profile |
| **Fixed Effects SE Method** | Model-Based |
| **Degrees of Freedom Method** | Containment |

| **Class Level Information** | | |
| --- | --- | --- |
| **Class** | **Levels** | **Values** |
| **Study** | 2 | 2 3 |

| **Dimensions** | |
| --- | --- |
| **Covariance Parameters** | 2 |
| **Columns in X** | 8 |
| **Columns in Z per Subject** | 1 |
| **Subjects** | 2 |
| **Max Obs per Subject** | 117 |

| **Number of Observations Used** | 198 |
| --- | --- |

| **Iteration History** | | | |
| --- | --- | --- | --- |
| **Iteration** | **Evaluations** | **-2 Res Log Like** | **Criterion** |
| **0** | 1 | 1638.24200068 |  |
| **1** | 1 | 1638.23499107 | 0.00000000 |

| Convergence criteria met. |
| --- |

| **Covariance Parameter Estimates** | | | | | |
| --- | --- | --- | --- | --- | --- |
| **Cov Parm** | **Subject** | **Estimate** | **Standard Error** | **Z Value** | **Pr > Z** |
| **UN(1,1)** | **Study** | 0.4238 | 5.4640 | 0.08 | 0.4691 |
| **Residual** |  | 260.59 | 26.8066 | 9.72 | <.0001 |

| **Fit Statistics** | |
| --- | --- |
| **-2 Res Log Likelihood** | 1638.2 |
| **AIC (Smaller is Better)** | 1642.2 |
| **AICC (Smaller is Better)** | 1642.3 |
| **BIC (Smaller is Better)** | 1639.6 |

| **Null Model Likelihood Ratio Test** | | |
| --- | --- | --- |
| **DF** | **Chi-Square** | **Pr > ChiSq** |
| 1 | 0.01 | 0.9333 |

| **Solution for Fixed Effects** | | | | | |
| --- | --- | --- | --- | --- | --- |
| **Effect** | **Estimate** | **Standard Error** | **DF** | **t Value** | **Pr > \|t\|** |
| **Intercept** | 60.4310 | 1.3048 | 1 | 46.32 | 0.0137 |
| **FutureFocus_Linear** | 9.4360 | 1.5387 | 189 | 6.13 | <.0001 |
| **FutureFocus_Quadr** | 0.3851 | 0.8241 | 189 | 0.47 | 0.6408 |
| **Favorability_Linear** | 6.3185 | 1.4858 | 189 | 4.25 | <.0001 |
| **Favorability_Quadr** | -0.9753 | 0.8514 | 189 | -1.15 | 0.2534 |
| **Attrib Disagree_Linear** | -6.1049 | 1.4371 | 189 | -4.25 | <.0001 |
| **Attrib Disagree_Quadr** | -1.3490 | 0.8103 | 189 | -1.66 | 0.0976 |
| **FutureFocus_Linear***  **Favorability__Linear** | -3.3729 | 1.9550 | 189 | -1.73 | 0.0861 |

| **Solution for Random Effects** | | | | | | |
| --- | --- | --- | --- | --- | --- | --- |
| **Effect** | **Study** | **Estimate** | **Std Err Pred** | **DF** | **t Value** | **Pr > \|t\|** |
| **Intercept** | **2** | -0.1526 | 0.6328 | 189 | -0.24 | 0.8097 |
| **Intercept** | **3** | 0.1526 | 0.6328 | 189 | 0.24 | 0.8097 |

| **Type 3 Tests of Fixed Effects** | | | | |
| --- | --- | --- | --- | --- |
| **Effect** | **Num DF** | **Den DF** | **F Value** | **Pr > F** |
| **Attrib Disagree_Linear** | 1 | 189 | 18.05 | <.0001 |
| **Attrib Disagree_Quadr** | 1 | 189 | 2.77 | 0.0976 |
| **FutureFocus_Linear** | 1 | 189 | 37.61 | <.0001 |
| **FutureFocus_Quadr** | 1 | 189 | 0.22 | 0.6408 |
| **Favorability_Linear** | 1 | 189 | 18.08 | <.0001 |
| **Favorability_Quadr** | 1 | 189 | 1.31 | 0.2534 |
| **FutureFocus_Linear ***  **Favorability_Linear** | 1 | 189 | 2.98 | 0.0861 |

The Mixed Procedure

| **Model Information** | |
| --- | --- |
| **Data Set** | WORK.TEMP |
| **Dependent Variable** | Intention to Change |
| **Covariance Structure** | Unstructured |
| **Subject Effect** | Study |
| **Estimation Method** | REML |
| **Residual Variance Method** | Profile |
| **Fixed Effects SE Method** | Model-Based |
| **Degrees of Freedom Method** | Containment |

| **Class Level Information** | | |
| --- | --- | --- |
| **Class** | **Levels** | **Values** |
| **Study** | 2 | 2 3 |

| **Dimensions** | |
| --- | --- |
| **Covariance Parameters** | 2 |
| **Columns in X** | 10 |
| **Columns in Z per Subject** | 1 |
| **Subjects** | 2 |
| **Max Obs per Subject** | 117 |

| **Number of Observations Used** | 198 |
| --- | --- |

| **Iteration History** | | | |
| --- | --- | --- | --- |
| **Iteration** | **Evaluations** | **-2 Res Log Like** | **Criterion** |
| **0** | 1 | 647.75288864 |  |
| **1** | 1 | 647.54382721 | 0.00000000 |

| Convergence criteria met. |
| --- |

| **Covariance Parameter Estimates** | | | | | |
| --- | --- | --- | --- | --- | --- |
| **Cov Parm** | **Subject** | **Estimate** | **Standard Error** | **Z Value** | **Pr > Z** |
| **UN(1,1)** | **Study** | 0.01458 | 0.04654 | 0.31 | 0.3770 |
| **Residual** |  | 1.3790 | 0.1426 | 9.67 | <.0001 |

| **Fit Statistics** | |
| --- | --- |
| **-2 Res Log Likelihood** | 647.5 |
| **AIC (Smaller is Better)** | 651.5 |
| **AICC (Smaller is Better)** | 651.6 |
| **BIC (Smaller is Better)** | 648.9 |

| **Null Model Likelihood Ratio Test** | | |
| --- | --- | --- |
| **DF** | **Chi-Square** | **Pr > ChiSq** |
| 1 | 0.21 | 0.6475 |

| **Solution for Fixed Effects** | | | | | |
| --- | --- | --- | --- | --- | --- |
| **Effect** | **Estimate** | **Standard Error** | **DF** | **t Value** | **Pr > \|t\|** |
| **Intercept** | 4.6336 | 0.1240 | 1 | 37.38 | 0.0170 |
| **FutureFocus_Linear** | 1.2970 | 0.1124 | 187 | 11.54 | <.0001 |
| **FutureFocus_Quadr** | -0.07973 | 0.06045 | 187 | -1.32 | 0.1888 |
| **Favorability_Linear** | 0.2025 | 0.1087 | 187 | 1.86 | 0.0640 |
| **Favorability_Quadr** | -0.03234 | 0.06206 | 187 | -0.52 | 0.6028 |
| **Attrib Disagree_Linear** | -0.2609 | 0.1082 | 187 | -2.41 | 0.0169 |
| **Attrib Disagree_Quadr** | -0.09397 | 0.05928 | 187 | -1.59 | 0.1146 |
| **FutureFocus_Linear ***  **Favorability_Linear** | -0.2985 | 0.1423 | 187 | -2.10 | 0.0373 |
| **Attrib Disagree_Linear ***  **FutureFocus_Linear** | -0.2343 | 0.1298 | 187 | -1.81 | 0.0726 |
| **Attrib Disagree_Quadr ***  **Favorability_Quadr** | 0.07052 | 0.04235 | 187 | 1.67 | 0.0976 |

| **Solution for Random Effects** | | | | | | |
| --- | --- | --- | --- | --- | --- | --- |
| **Effect** | **Study** | **Estimate** | **Std Err Pred** | **DF** | **t Value** | **Pr > \|t\|** |
| **Intercept** | **2** | 0.05686 | 0.1065 | 187 | 0.53 | 0.5942 |
| **Intercept** | **3** | -0.05686 | 0.1065 | 187 | -0.53 | 0.5942 |

| **Type 3 Tests of Fixed Effects** | | | | |
| --- | --- | --- | --- | --- |
| **Effect** | **Num DF** | **Den DF** | **F Value** | **Pr > F** |
| **FutureFocus_Linear** | 1 | 187 | 133.24 | <.0001 |
| **FutureFocus_ Quadr** | 1 | 187 | 1.74 | 0.1888 |
| **Favorability_Linear** | 1 | 187 | 3.47 | 0.0640 |
| **Favorability_Quadr** | 1 | 187 | 0.27 | 0.6028 |
| **Attrib Disagree_Linear** | 1 | 187 | 5.81 | 0.0169 |
| **Attrib Disagree_Quadr** | 1 | 187 | 2.51 | 0.1146 |
| **FutureFocus_Linear ***  **Favorability_Linear** | 1 | 187 | 4.40 | 0.0373 |
| **Attrib Disagree_Linear ***  **FutureFocus_Linear** | 1 | 187 | 3.26 | 0.0726 |
| **Attrib Disagree_Quadr ***  **Favorability_Quadr** | 1 | 187 | 2.77 | 0.0976 |
